# Supplementary material for: Uncovering the dynamics of precise repair at CRISPR/Cas9-induced double-strand breaks
Source: Nat Commun. 2024 Jun 14;15:5096. doi: 10.1038/s41467-024-49410-x (PMC11178868; doi:10.1038/s41467-024-49410-x)
Supplement: Supplementary file 1 — Supplementary Information [file 41467_2024_49410_MOESM1_ESM.pdf]

Supplementary Figures and Tables

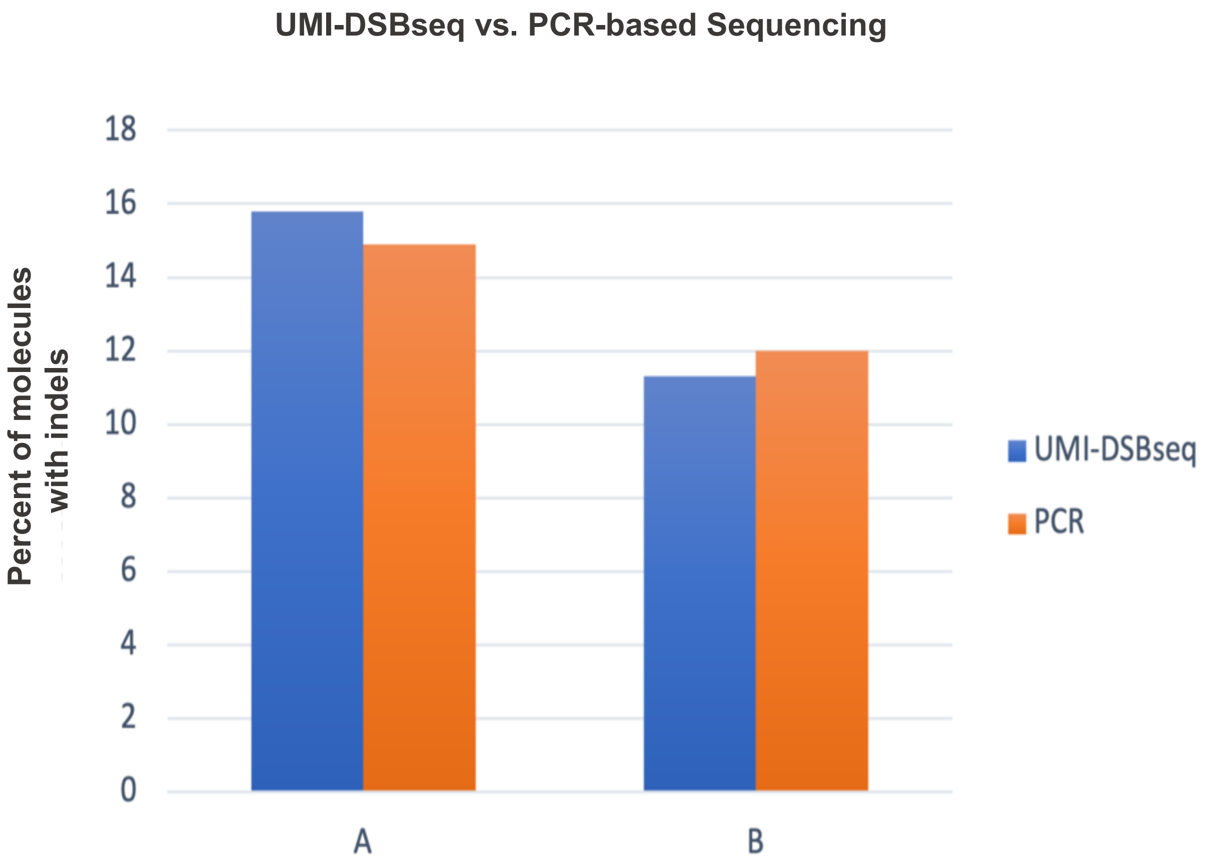

Figure S1. Comparison of the frequency of indels estimated using UMI-DSBseq or direct PCR-based amplicon sequencing. Illumina libraries containing *Psy1* sequences, were prepared by either UMI-DSBseq (blue) or via sequencing of amplicons with primers flanking the DSB site (PCR-orange). The percent of molecules with indels out of the total molecules (excluding unrepaired DSBs for UMI-DSB-seq) is shown in two replicas (A and B). Data can be found in Source Data Figure S1.

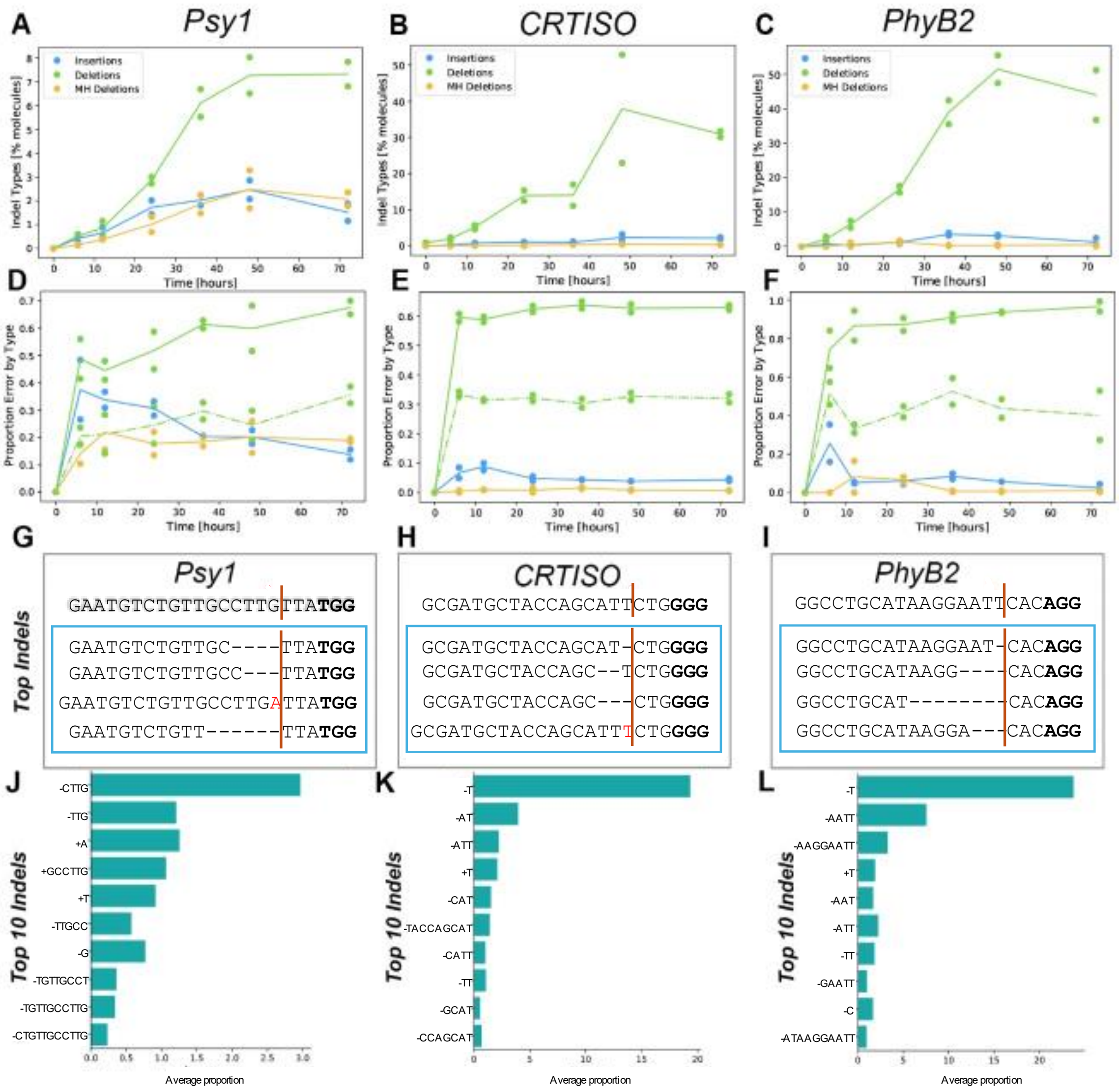

Figure S2. Footprints of error-prone repair at 3 targets in tomato. A-C) Percent of different type of indels out of total molecules for (A) *Psy1*, (B) *CRTISO* and (C) *PhyB2*. Insertions are shown in blue, deletions in green and deletions with 2 or more bp of microhomology in yellow. D-F) Proportion of each type out of total indels, during the time course from 0 to 72 hours, for (D) *Psy1*, (I) *CRTISO* and (F) *PhyB2.* The proportion of the top indel (a deletion for all targets) is further indicated with a dashed line. Dots represent each duplicate and lines represent the mean of the replicates. G-I) Sequence of each target with PAM indicated in bold and red line for the expected cut site. Top indels are shown in the box, ranked by order of abundance for (G) *Psy1*, (H) *CRTISO*, and (I) *PhyB2.* J-L) Average proportion of the top 10 indels for (J) *Psy1*, (K) *CRTISO*, and (L) *PhyB2*.

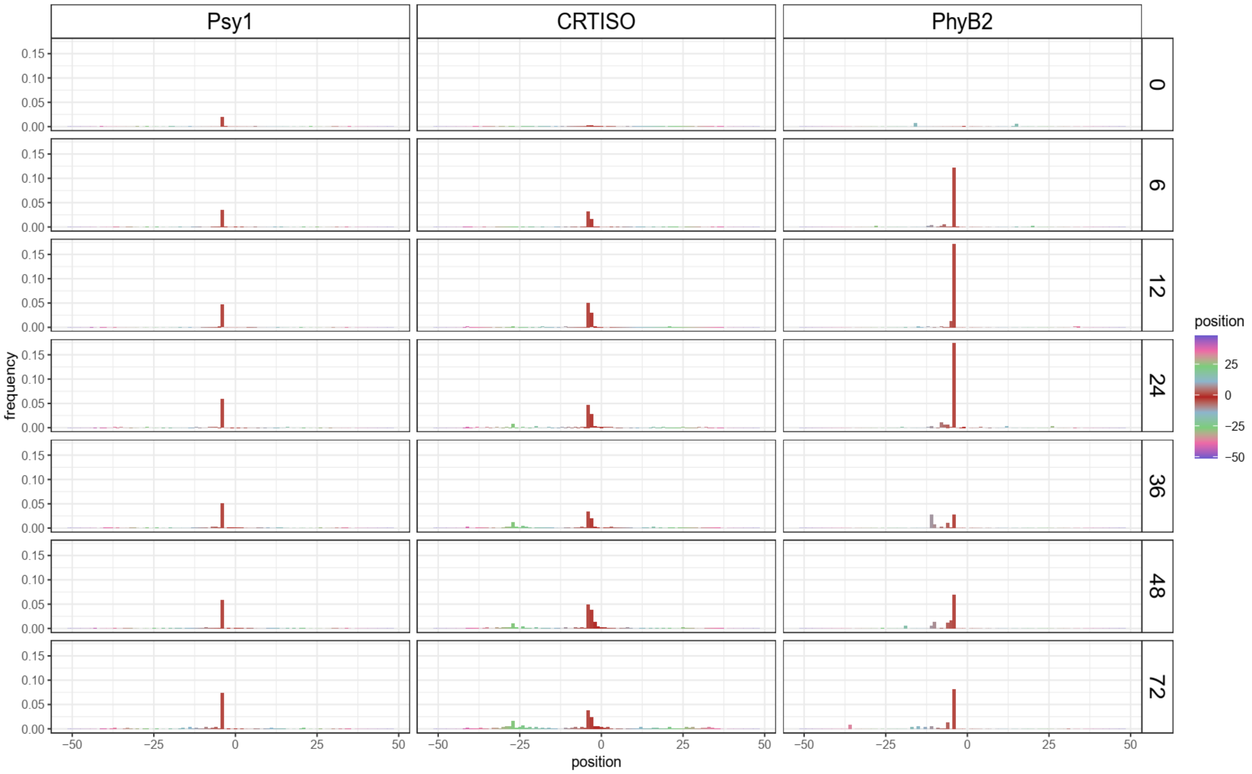

Figure S3. DSBs captured in time-courses over time (0, 6, 12, 24, 36, 48 and 72hours shown in different vertical facets) plotted by position of the captured end. Data can be found in Source Data.

Table S1. Estimates of the Rates and Induction parameters for the 3-state model of DSB Repair with transformation time set as time 0h and first sampling at 30 minutes.

Target	Process	Rate[a] (proportion of total event/hour)	CI [1-99%][a,c]	Flow[b] after 72h (proportion of total molecules)	CI [1-99%] [b,c]	P.value
Psy1	Cutting	0.0092	0.0065-0.4922	0.5729	0.4046-27.9219	<0.01
	Repair-error	0.0335	0.0265-0.0403	0.151	0.1355-0.1662	<0.01
	Precise Repair	0.0791	0.0395-6.8544	0.3562	0.1852-27.518	<0.01
	Error	0.2052	0.1715-0.2377	/	/	<0.01
	U (uncut fraction)	0	0-0.0992	/	/	0.95
	r (induction speed)	40.4375	2.3747-786.794	/	/	<0.01
	d (induction decay)	0	0-0	/	/	<0.01
	Repair accuracy	/	/	0.7023	0.5517-0.9943	<0.01
CRTISO	Cutting	0.0175	0.0124-0.0259	0.878	0.5407-1.3316	<0.01
	Repair-error	0.0777	0.0606-0.0976	0.435	0.3579-0.5131	<0.01
	Precise Repair	0.0676	0-0.1542	0.3786	0-0.8617	0.11
	Error	0.32	0.2271-0.3881	/	/	<0.01
	U (uncut fraction)	0	0-0.0992	/	/	0.95
	r (induction speed)	17.5338	5.436-52.7849	/	/	<0.01
	d (induction decay)	0	0-0.0048	/	/	<0.01
	Repair accuracy	/	/	0.4653	0-0.7065	0.11
PhyB2	Cutting	0.072	0.0441-0.2409	0.913	0.5756-3.6319	<0.01
	Repair-error	0.0864	0.073-0.1064	0.527	0.4931-0.5685	<0.01
	Precise Repair	0.0589	0.0035-0.5863	0.3591	0.0213-3.0766	0.01
	Error	0.0817	0.044-0.1252	/	/	<0.01
	U (uncut fraction)	0.4045	0-0.4458	/	/	0.07
	r (induction speed)	6.1986	3.2012-20.0938	/	/	<0.01
	d (induction decay)	0	0-0.0081	/	/	<0.01
	Repair accuracy	/	/	0.4052	0.0362-0.8537	0.01

[a]Rates are reported as the number of events per molecule per hour. [b]The flow is reported as the proportion of molecules that experienced the specific event at the end of the experiment. [c]Confidence intervals (CI) are reported as the 1% and 99% percentiles of the estimates obtained from 100 stratified bootstraps of the data, while p.values as the proportion of bootstraps with value smaller or equal than 0 (one-sided test). When none of the 100 bootstraps had value equal to 0 we reported p.values as <0.01. The induction curve is modeled as a logistic increase in activity of the RNPs with speed *r*, a fraction *U* of cells upon which the RNPs do not cut DNA, and a decay *d*. An error rate *e* describes the proportion of DSBs which show DSB ends not coinciding with the expected position (see *Determining the parameters of the induction curve).* Repair accuracy is computed as the proportion of DSBs repaired precisely over all repaired DSBs at the end of the time course (72h).

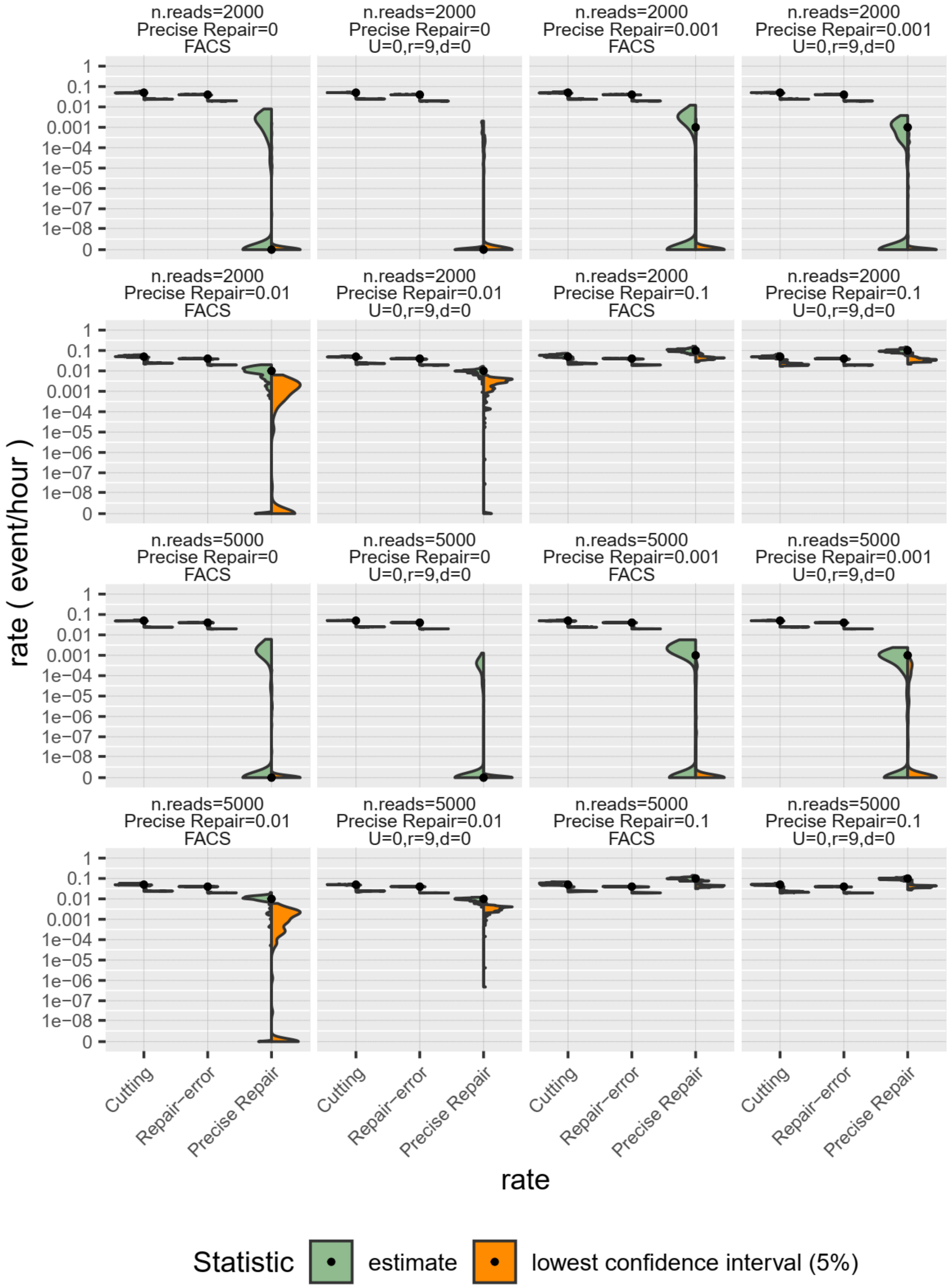
n.reads=2000
Precise Repair=0
FACS
n.reads=2000
Precise Repair=0
U=0,r=9,d=0
n.reads=2000
Precise Repair=0.001
FACS
n.reads=2000
Precise Repair=0.001
U=0,r=9,d=0
n.reads=2000
Precise Repair=0.01
FACS
n.reads=2000
Precise Repair=0.01
U=0,r=9,d=0
n.reads=2000
Precise Repair=0.1
FACS
n.reads=2000
Precise Repair=0.1
U=0,r=9,d=0
n.reads=5000
Precise Repair=0
FACS
n.reads=5000
Precise Repair=0
U=0,r=9,d=0
n.reads=5000
Precise Repair=0.001
FACS
n.reads=5000
Precise Repair=0.001
U=0,r=9,d=0
n.reads=5000
Precise Repair=0.01
FACS
n.reads=5000
Precise Repair=0.01
U=0,r=9,d=0
n.reads=5000
Precise Repair=0.1
FACS
n.reads=5000
Precise Repair=0.1
U=0,r=9,d=0
1
0.1
0.01
0.001
1e−04
1e−05
1e−06
1e−07
1e−08
0
rate (event/hour)
Cutting
Repair−error
Precise Repair
rate
Statistic
estimate
lowest confidence interval (5%)

Figure S4. Simulations and testing of the 3 state model. Violin plots showing the distributions of the point estimates (green) and lowest confidence intervals (orange) of rate parameters for 100 simulations. Simulated values shown as a black dot. Different values of precise repair (0,0.1,0.01 or 0.001 events/hour) and numbers of reads (2000 and 5000) were simulated. Two sets of representative induction curve parameters shown: a curve with no decay (r=9 U=0 and d=0) and a curve with very rapid induction and a slow decay (U=0.02529, r=85.9774, d=0.00263), which was estimated from the FACS as described below and used in Fig. S9, Fig.S10, Fig.S14. Simulated parameters were generally selected to reflect those observed in our data. Note that across all conditions, very little false positives are observed when precise repair is absent, i.e. the orange distribution is centered around 0 when Precise Repair=0; and for those, the point estimate is always lower than 0.01, which is more than 5 folds lower than the point estimates for *Psy1* and *Phyb2*. Simulations were generated using the code in https://github.com/fabrimafe/DSBtimecourse/tree/natcom2024 and deposited at https://doi.org/10.5281/zenodo.11218185.

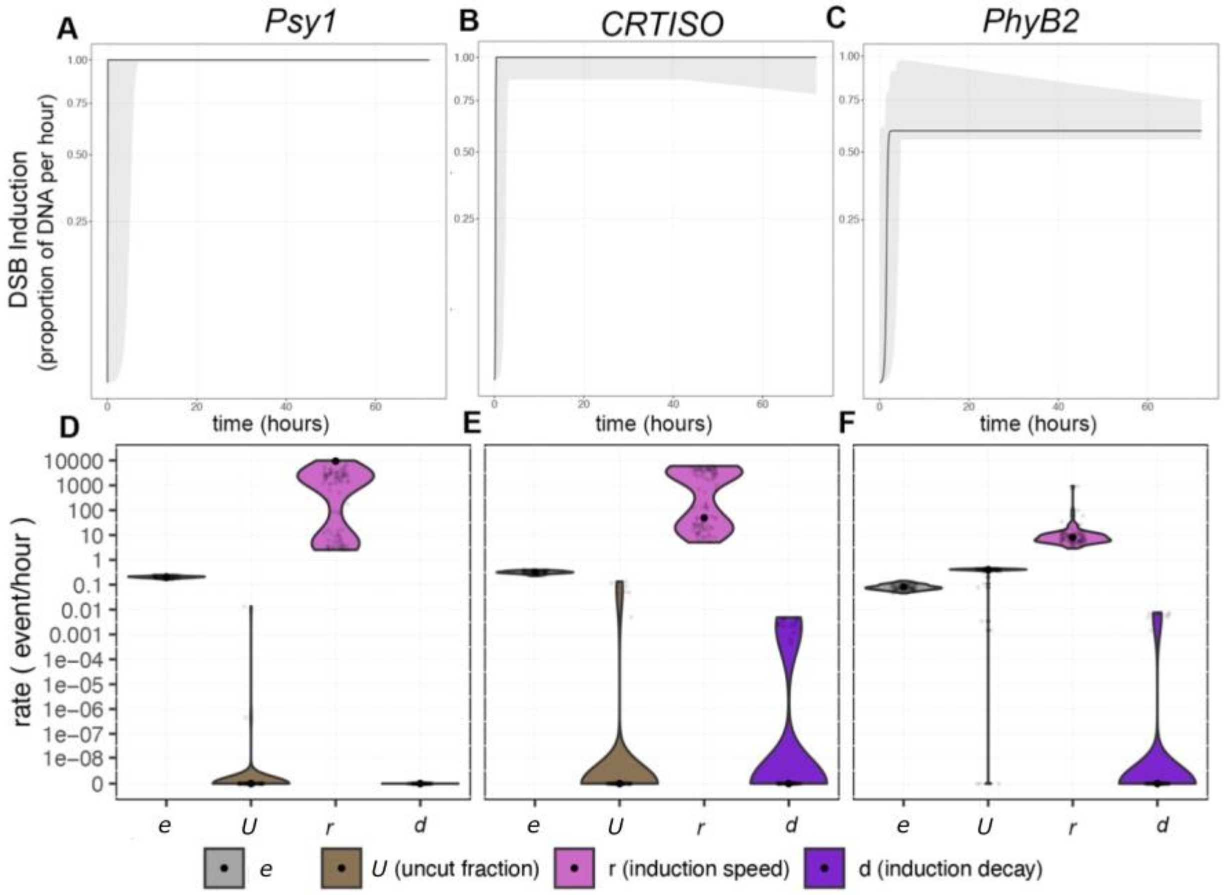

Figure S5. Induction Curve predicted by 3-state model. A-C) Induction curves with confidence intervals from the bootstrap indicated in gray shading D-F) Estimates for *e* (the proportion of unexpected DSBs over total DSBs, potentially introduced by the experimental processing or sequencing which result in some DSBs to end at positions different than that corresponding to the expected Cas9 cutting site) and the three parameters of the induction curve, uncut fraction (*U*), induction speed (*r*), and induction decay (*d*)(see Methods). The uncut fraction (*U*) represents the estimated proportion of intact DNA molecules which are not being cut throughout the time-course. The induction speed (*r*) determines the slope of the induction curve, i.e. how rapid is the increase in cutting rate after the delivery of the RNPs, while the induction decay determines the rate at which the induction of DSBs decrease over time, possibly as a result of depletion of RNPs. D-F) The smoothed distribution of the estimates obtained through the bootstrap procedure is shown as a violin plot. The estimate obtained from the original (before bootstrapping) data is shown as a black point at (A,D) *Psy1*, (B,E) *CRTISO* and (C,F) *PhyB2*. See related Figure 4, Table1, Table S1.

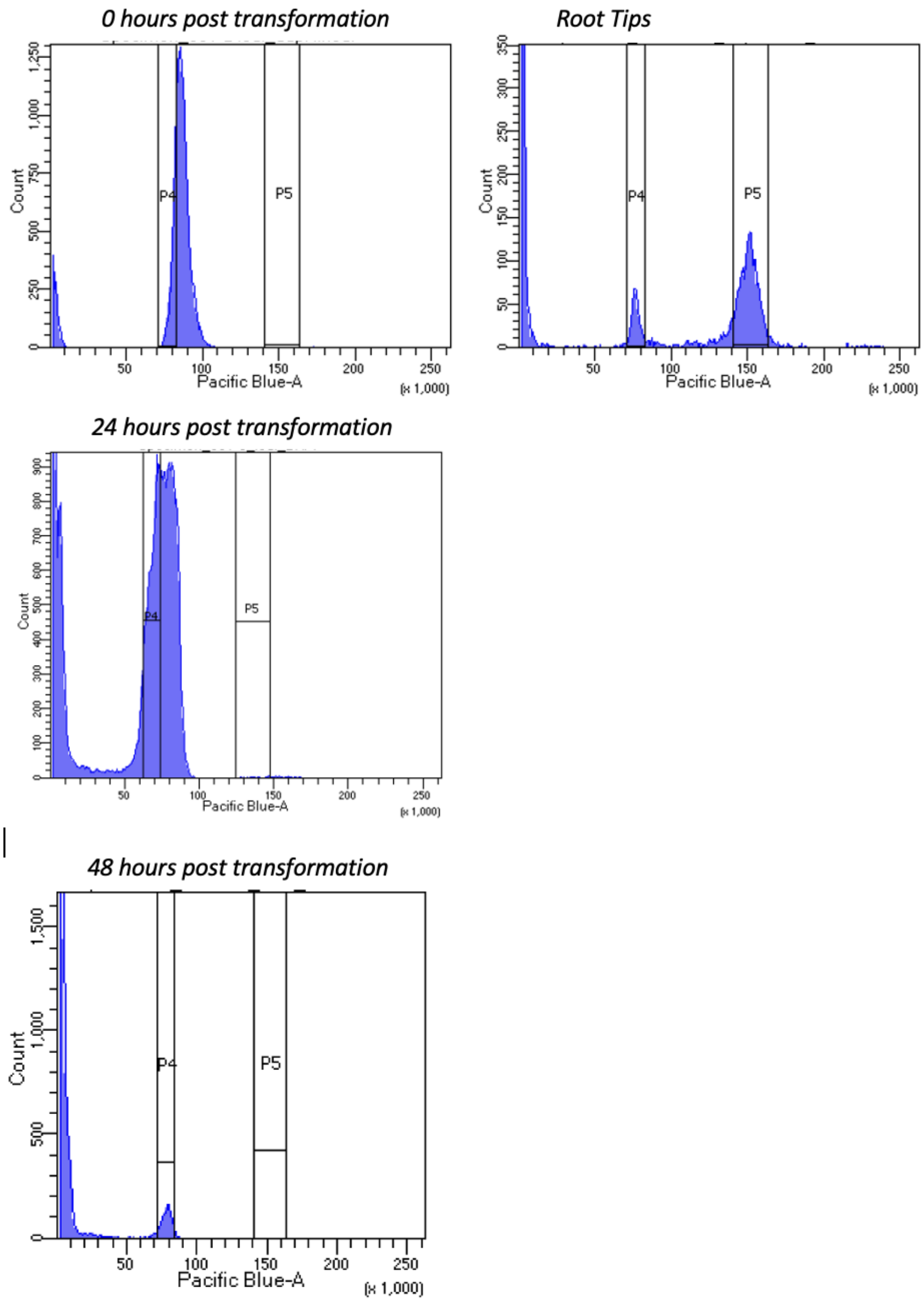

Figure S6. Fluorescence assisted cell sorting (FACS) analysis of cell cycle phase at 0, 24 and 48 hours post transformation. Root tips are used as a control for cycling cells. P4 represents cell cycle phase G1 and P5 represents G2. Counts of cells are shown on the y-axis, fluorescence intensity on the x-axis. Flow cytometry data are deposited in https://zenodo.org/doi/10.5281/zenodo.11255716.

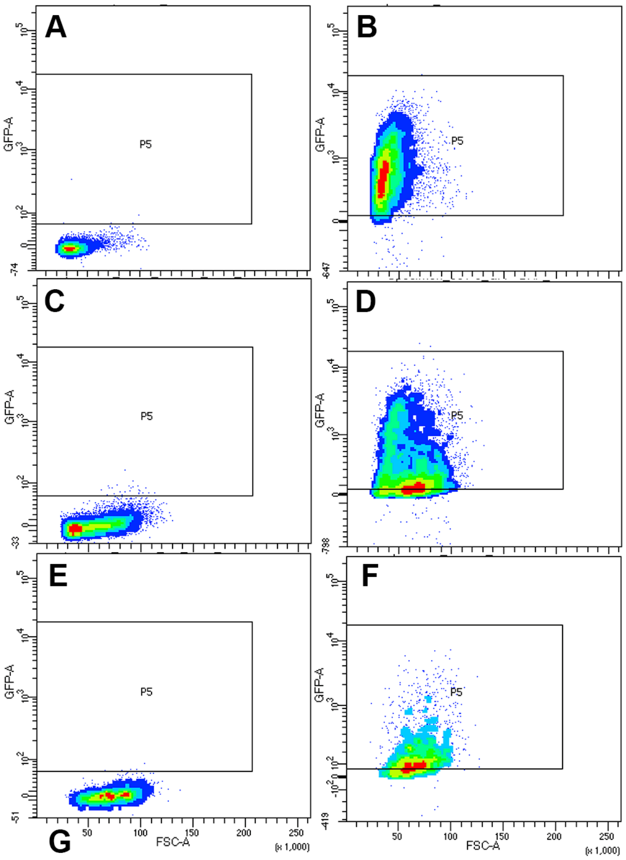

G

Time	Population	Cas9 RNP	*% GFP+*	Cas9-GFP RNP	*% GFP +*
**0h**	*G1 nuclei*	3571	0.16%	4432	97.42%
	GFP +	6		4318	
**24h**	*G1 nuclei*	4572	0.61%	4358	92.97%
	GFP +	28		4052	
**48h**	*G1 nuclei*	833	0.60%	1094	89.9%
	GFP +	5		984	

Figure S7. FACS analysis of transformation efficiency. Cells transformed with RNPs either with Cas9 (control) or Cas9 fused to GFP, where sorted immediately following transformation at 24 hours and at 48 hours. Nuclei were extracted and stained with DAPI. Gating was first done to select for round, vital nuclei followed by quantification of GFP fluorescence. A,C and E) Control cells transformed with Cas9 RNPs; B,D and F) GFP-Cas9 transformed cells; A,B) Cells evaluated immediately after transformation; C,D) 24 hours after transformation; E,F) 48 hours following transformation; G) The total nuclei counted with and without GFP along all the experiment. Percent of GFP positive nuclei is indicated for all the time points in the table and in all figures within gate channel P5. Flow cytometry data are deposited in https://zenodo.org/doi/10.5281/zenodo.11255716.

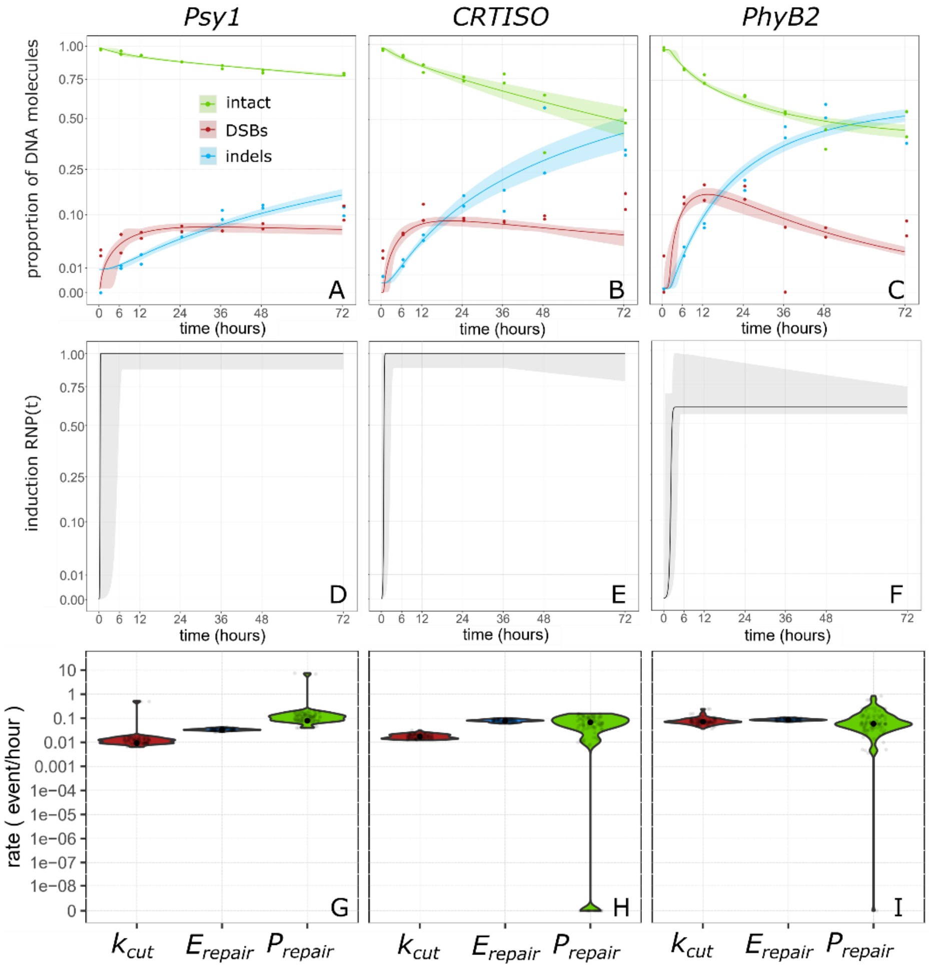

Figure S8. Kinetic model with 3 states including transformation time. A-C) Fit of the 3-state model to the data at *Psy1* (A), *CRTISO* (B) and PhyB2 (C). Intact molecules are shown in green, DSB in red, indels in blue. Confidence Intervals represented in shading and calculated from 100 iterations of the bootstrap. Observed experimental data represented as dots. D-F) Double Strand Break induction curves for *Psy1* (D), *CRTISO* (E) and *PhyB2* (F)*.* Confidence intervals are shown as gray shadings and calculated from 100 bootstraps of the data. G-I) Rate constants estimated in terms of number of events per hour per molecule for *Psy1* (G), *CRTISO* (H) and *PhyB2* (I). The smoothed distribution of the estimates obtained through the bootstrap procedure is shown as a violin plot. Grey points represent 100 instances of stratified bootstrap. The estimate obtained from the original (before bootstrapping) data is shown as a black point. Transformation time was accounted for by setting time 0h at the time of transformation and the first sampling at 30 minutes after transformation.

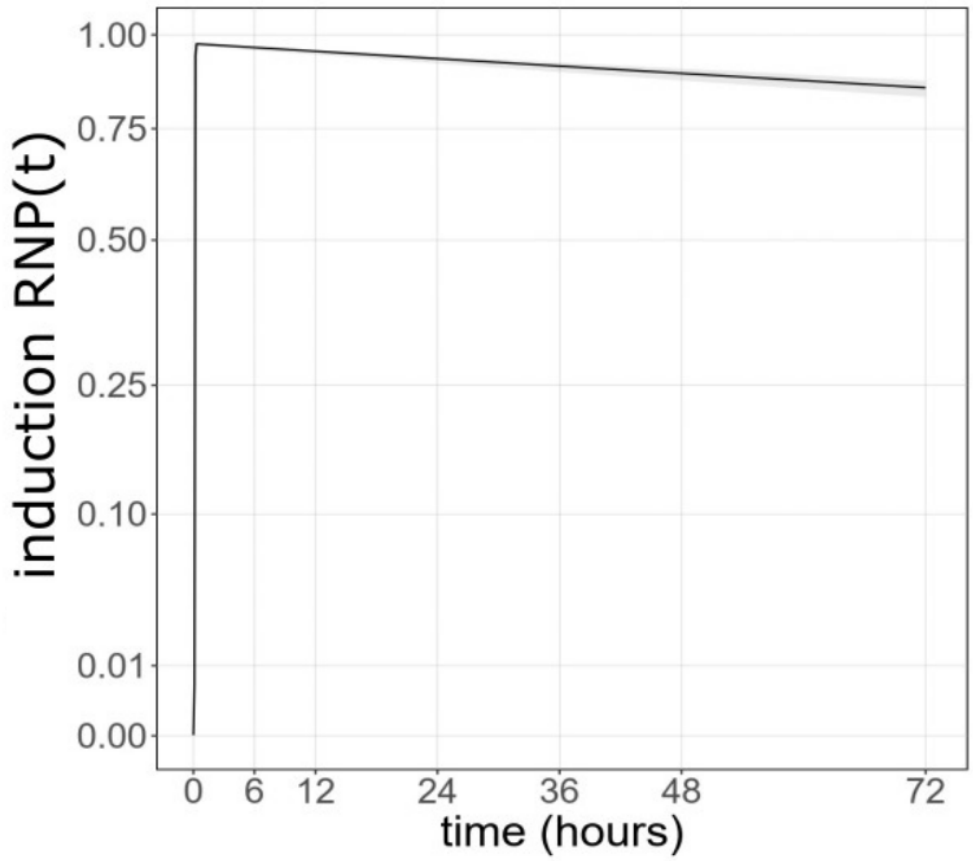

Figure S9. Induction curve estimated from FACS. The curve was estimated from Fig.S7G, fitting the Cas9-GFP RNP proportions to the induction curve formula used throughout the manuscript, using a maximum binomial likelihood. The confidence intervals, shown in grey, are estimated using a 5% likelihood-ratio based confidence interval. The estimated parameters are U=0.02529, r=85.9774, d=0.00263.

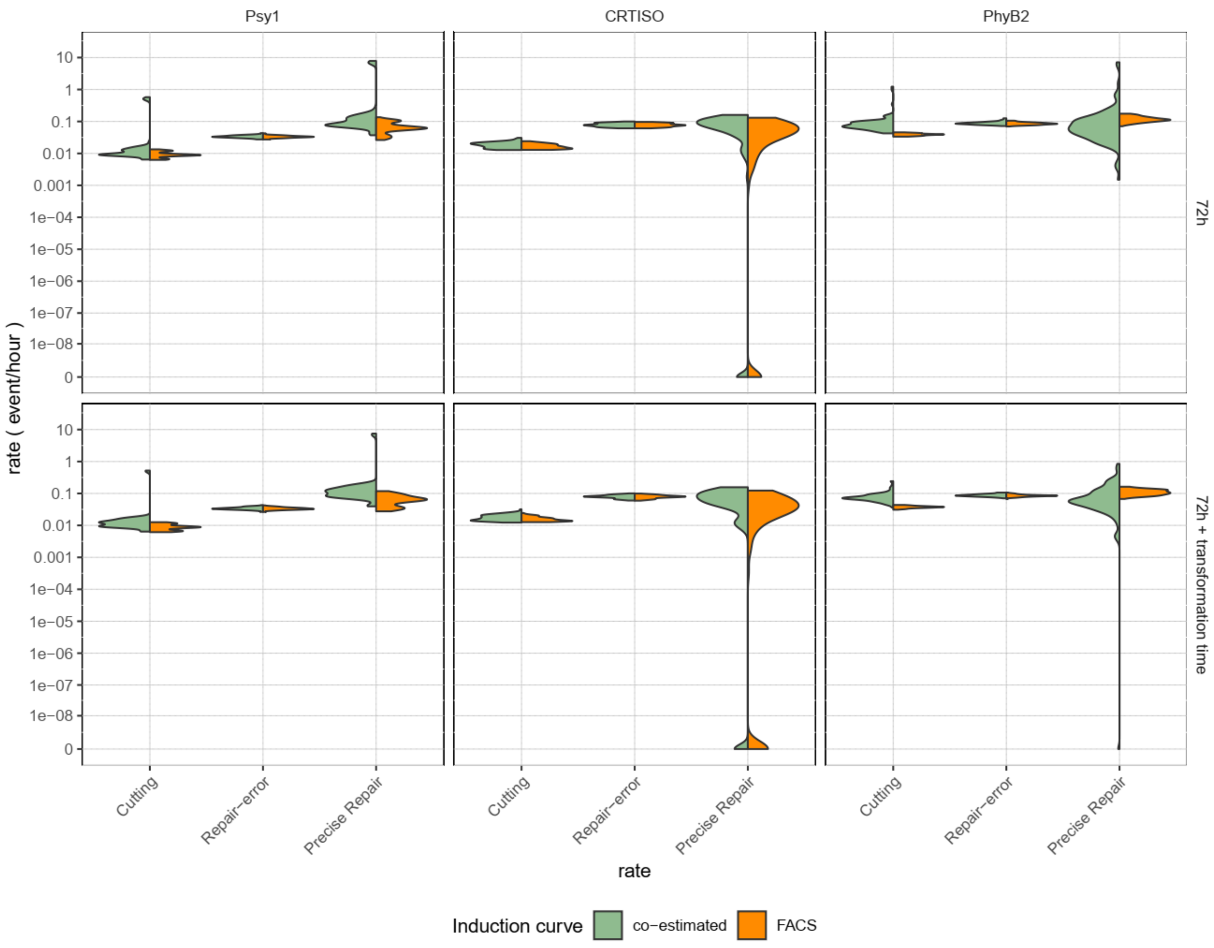

Figure S10. Comparison of estimates when co-estimating induction curve and repair parameters (green) or using the induction curve estimated from FACS data (orange) for the 3-state model. Violin plots represent the bootstrap estimates for the two methods. Note how fixing the induction curve reduces the number of parameters and the uncertainty, especially for PhyB2.

Target	Process	Rate[a] (proportion of total event/hour)	CI [1-99%][a,c]	Flow[b] after 72h (proportion of total molecules)	CI [1-99%] [b,c]	P.value
Psy1	Cutting	0.009	0.0063-0.0126	0.5145	0.4928-0.4928	<0.01
	Repair-error	0.0334	0.0276-0.0394	/	0.1534-0.1534	<0.01
	Precise Repair	0.0646	0.0271-0.1121	/	0.2757-0.2757	<0.01
	Error	0.2044	0.1659-0.2409	/	/	<0.01
	U (uncut fraction)	0.0253	0.0253-0.0253	/	/	/
	r (induction speed)	85.9775	85.9775-85.9775	/	/	/
	d (induction decay)	0.0026	0.0026-0.0026	/	/	/
	Repair accuracy	0.6591	0.4668-0.7771	/	0.6425-0.6425	<0.01
CRTISO	Cutting	0.0162	0.013-0.0234	0.7483	0.7131-0.7131	<0.01
	Repair-error	0.0775	0.0618-0.0953	/	0.4368-0.4368	<0.01
	Precise Repair	0.0408	0-0.1153	/	0.2205-0.2205	0.06
	Error	0.3159	0.2317-0.3872	/	/	<0.01
	U (uncut fraction)	0.0253	0.0253-0.0253	/	/	/
	r (induction speed)	85.9775	85.9775-85.9775	/	/	/
	d (induction decay)	0.0026	0.0026-0.0026	/	/	/
	Repair accuracy	0.345	0-0.6514	/	0.3355-0.3355	0.06
PhyB2	Cutting	0.0387	0.0335-0.0455	1.4038	1.4102-1.4102	<0.01
	Repair-error	0.0848	0.0737-0.105	/	0.5788-0.5788	<0.01
	Precise Repair	0.1131	0.0799-0.1683	/	0.7861-0.7861	<0.01
	Error	0.0868	0.0512-0.1453	/	/	<0.01
	U (uncut fraction)	0.0253	0.0253-0.0253	/	/	/
	r (induction speed)	85.9775	85.9775-85.9775	/	/	/
	d (induction decay)	0.0026	0.0026-0.0026	/	/	/
	Repair accuracy	0.5714	0.477-0.6583	/	0.576-0.576	<0.01

Table S2. Estimates of the Rates and Induction parameters for the 3-state model of DSB Repair with induction parameters fixed to those estimated from FACS data (U=0.0253, r=85.9775, d=0.0026, Fig. S9).

[a]Rates are reported as the number of events per molecule per hour. [b]The flow is reported as the proportion of molecules that experienced the specific event at the end of the experiment. [c]Confidence intervals (CI) are reported as the 1% and 99% percentiles of the estimates obtained from 100 stratified bootstraps of the data, while p.values as the proportion of bootstraps with value smaller or equal than 0 (one-sided test). When none of the 100 bootstraps had value equal to 0 we reported p.values as <0.01.

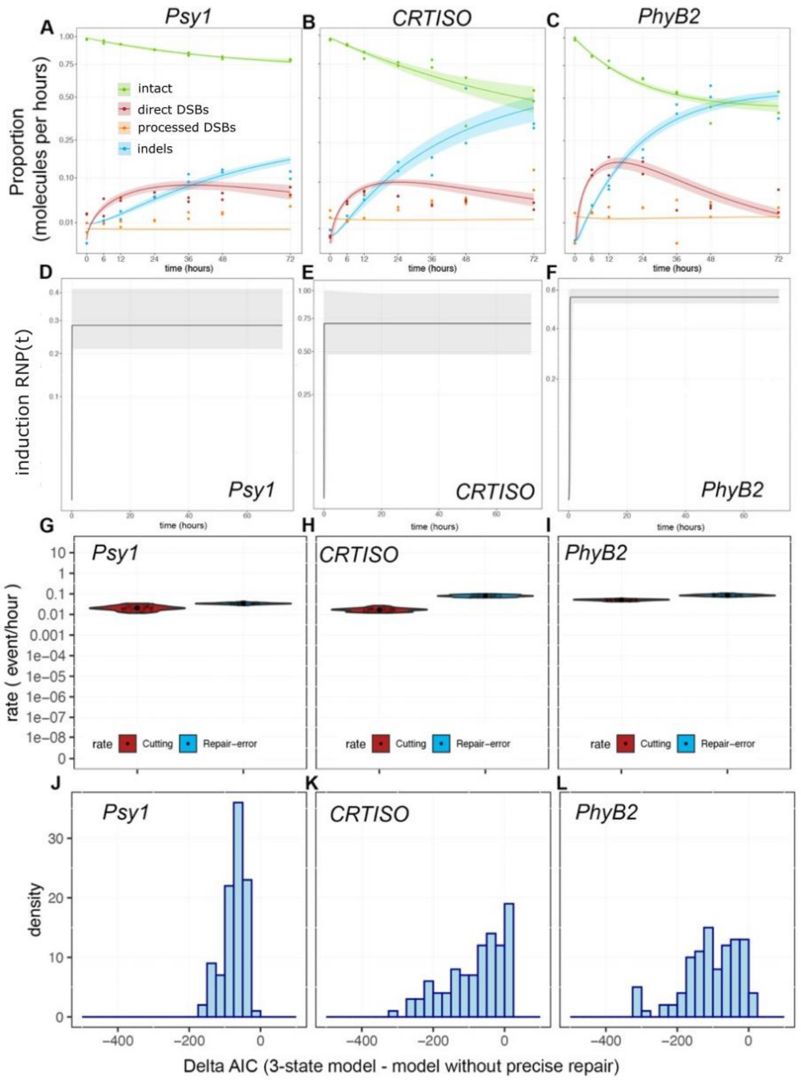

Figure S11. Kinetic model with no precise repair. A-C) Predicted fit of the 3-state model (lines) at *Psy1* (A), *CRTISO* (B), and *PhyB2* (C) for Intact molecules (green), DSB (red), indels (blue) and processed DSB (orange). Observed experimental data are represented as dots. D-F) Induction curves for *Psy1* (D), *CRTISO* (E) and *PhyB2* (F). Confidence intervals are shown as gray shadings and calculated from 100 bootstraps of the data. . G-I) Rate constants estimated in terms of number of events per hour per molecule for at *Psy1* (G), *CRTISO* (H), and *PhyB2* (I) in terms of number of events per hour per molecule. The smoothed distribution of the estimates obtained through the bootstrap procedure is shown as a violin plot. The estimate obtained from the original data (before bootstrapping) is shown as a black point. Grey points represent 100 instances of stratified bootstrap. J-L) Difference in AIC (Delta AIC, where AIC stands for Akaike Information Criterion) between the 3-state model with and without perfect repair for Psy1 (J), *CRTISO* (K), and *PhyB2* (L). AIC takes into account the likelihood of the models as well as its complexity, i.e. the number of parameters in the model, to establish which model is best supported by the data. Delta AIC values higher than 0 indicate higher support for the simpler model (3-state model without perfect repair), while negative values support the more complex model (3-state model). A Delta AIC of ~-6 corresponds to a relative likelihood of 0.05, indicating strong support for the more complex model (see related Figure 4, Table S2).

Target	Rate	Rate Constant[a] (proportion events/hour)	CI[a,c] (1%)	CI[a,c] (99%)	Flow after 72h[b] (proportion)	CI[b,c] (1%)	CI[b,c] (99%)
Psy1	Cutting	0.0205	0.0117	0.0334	0.2192	0.1942	0.2331
	Repair-error	0.0341	0.0278	0.0416	0.1581	0.1433	0.1705
	e (inac.DSB)	0.2035	0.1631	0.2406	/	/	/
	U (induction)	0.7160	0.1902	0.7541	/	/	/
	r (induction)	27778.8235	0.0000	46083.6335	/	/	/
	decay (induction)	0.0000	0.0000	0.0000	/	/	/
PhyB2	Cutting	0.0504	0.0422	0.0584	0.5453	0.5163	0.5805
	Repair-error	0.0850	0.0731	0.1046	0.5250	0.4941	0.5598
	e (inac.DSB)	0.0809	0.0469	0.1364	/	/	/
	U (induction)	0.4395	0.3944	0.4731	/	/	/
	r (induction)	32.0782	12.3336	61983.8014	/	/	/
	decay (induction)	0.0000	0.0000	0.0000	/	/	/
CRTISO	Cutting	0.0171	0.0122	0.0254	0.5010	0.4055	0.5816
	Repair-error	0.0803	0.0661	0.0976	0.4456	0.3633	0.5219
	e (inac.DSB)	0.3131	0.2281	0.3824	/	/	/
	U (induction)	0.2914	0.0000	0.4850	/	/	/
	r (induction)	30886.4754	0.0000	74548.3365	/	/	/
	decay (induction)	0.0000	0.0000	0.0053	/	/	/

Table S3. Rate Constants estimated for kinetic model without precise repair.

Estimates for the rate constants and the three parameters of the induction curve, uncut fraction (*U*), induction speed (*r*), and induction decay (*d*)(see Methods). **[a]**Rates are reported as the number of events per molecule per hour. **[b]**The flow is reported as the proportion of molecules that experienced that event at the end of the experiment. **[c]**Confidence intervals (CI) are reported as the 1% and 99% percentiles of the estimates obtained from 100 stratified bootstraps of the data.

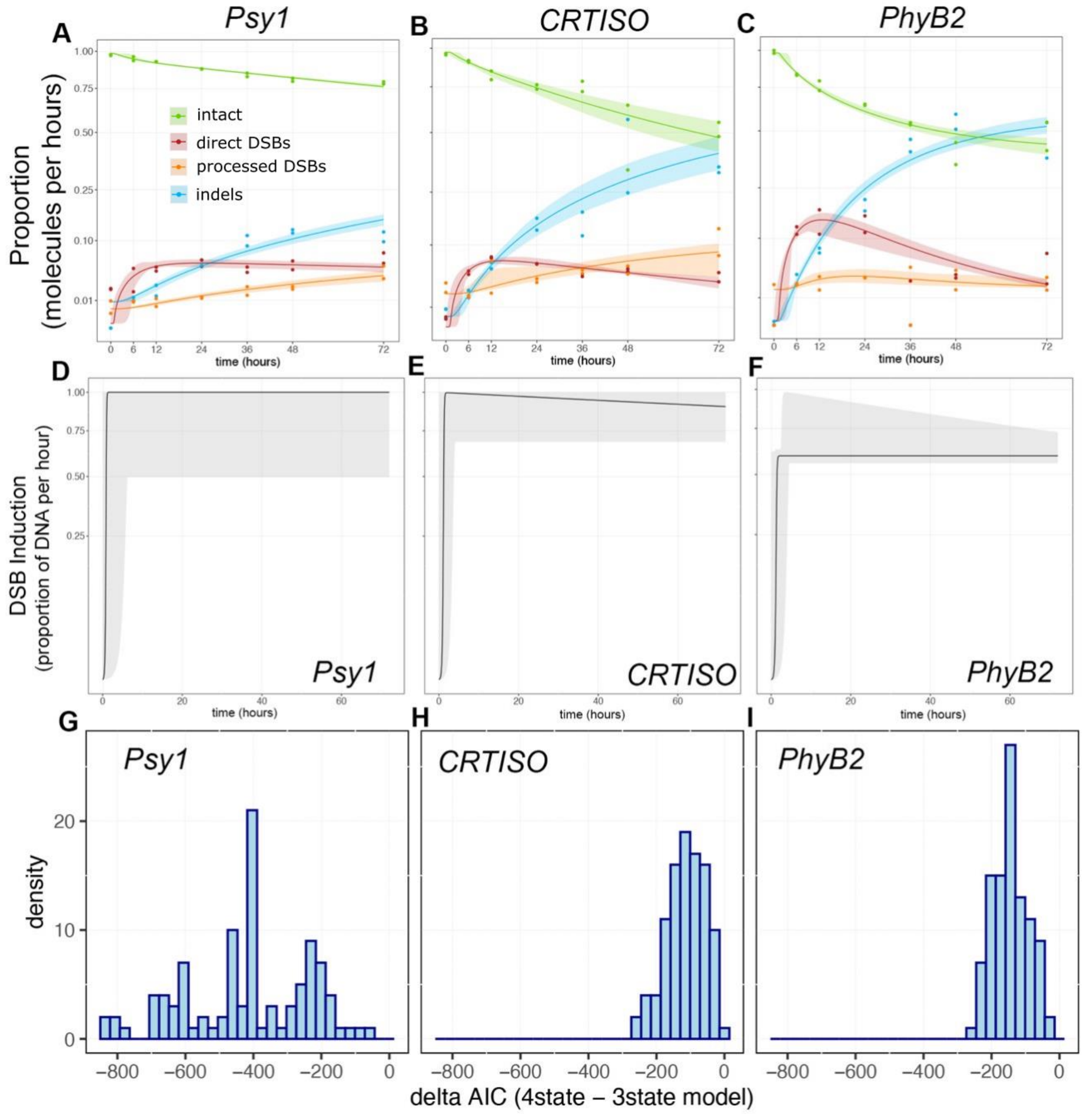

Figure S12.**The 4-state model of DSB induction and repair.** A-C) Fit of the model to the data at *Psy1* (A), *CRTISO* and *PhyB2* (C). Intact molecules are shown in green, DSB in red, indels in blue and processed DSB in orange. Confidence intervals are represented in shading and calculated from 100 iterations of the bootstrap. D-F) Induction curves with confidence intervals from the bootstrap indicated in gray shading at *Psy1* (D), *CRTISO* (E), and *PhyB2* (F). G-I) Difference in AIC (Delta AIC, where AIC stands for Akaike Information Criterion) between the 4-state model and the 3-state model at *Psy1* (G), *CRTISO* (H), and *PhyB2* (I). AIC takes into account the likelihood of the models as well as its complexity, i.e. the number of parameters in the model, to establish which model is best supported by the data. Delta AIC values higher than 0 indicate higher support for the simpler model (3-state model), while negative values support the more complex model (4-state model). A Delta AIC of ~-6 corresponds to a relative likelihood of 0.05, indicating strong support for the more complex model (see related Figure 6, Table S4).

Target	Process	Rate Constant[a]	CI (bootstrap percentile)				Flow at 72h	CI (bootstrap percentile)				
			1%	5%	95%	99%		1%	5%	95%	99%	P.value
Psy1	K_{cut}	0.0104	0.0087	0.0088	0.35	0.3839	0.6411	0.4412	0.5412	19.7274	21.7711	<0.01
	$K_{processing}$	0.009	0.0075	0.008	0.0109	0.0127	0.0316	0.0252	0.0278	0.0377	0.0436	<0.01
	E_{direct}	0.0421	0.0353	0.037	0.0473	0.0502	0.1477	0.1323	0.1369	0.1572	0.1612	<0.01
	$E_{processed}$	0	0	0	0	0	0	0	0	0	0	1
	P_{direct}	0.118	0.0638	0.0844	5.9301	6.6985	0.4133	0.218	0.3098	19.385	21.4026	<0.01
	$P_{processed}$	0	0	0	0.0123	0.0192	0	0	0	0.0117	0.0194	0.76
	U	0	0	0	0.2667	0.4904	/	/	/	/	/	0.80
	r	15.4794	2.2269	2.2402	2309.488	2878.201	/	/	/	/	/	0
	d	0	0	0	0	0.0004	/	/	/	/	/	0.96
	Precise Repair	/	/	/	/	/	0.4133	0.2201	0.3098	19.385	21.4026	<0.01
	Repair-error	/	/	/	/	/	0.1477	0.1323	0.1369	0.1572	0.1612	<0.01
	Repair accuracy	/	/	/	/	/	0.7367	0.5909	0.6774	0.9922	0.9931	<0.01
CRTISO	K_{cut}	0.0178	0.0136	0.0138	0.0276	0.0336	0.812	0.5487	0.5621	1.1347	1.3952	<0.01
	$K_{processing}$	0.0183	0.0127	0.0142	0.0241	0.1536	0.0661	0.0477	0.052	0.0848	0.5587	<0.01
	E_{direct}	0.1138	0.0926	0.095	0.1319	0.1356	0.4101	0.3402	0.3449	0.4772	0.4897	<0.01
	$E_{processed}$	0	0	0	0	0	0	0	0	0	0	1
	P_{direct}	0.0836	0	0	0.1733	0.1872	0.3012	0	0	0.6244	0.6551	0.26
	$P_{processed}$	0	0	0	0.0132	0.2741	0	0	0	0.0278	0.5367	0.71
	U	0	0	0	0.2153	0.2741	/	/	/	/	/	0.74
	r	11.4862	4.5798	6.4035	474.3362	1070.389	/	/	/	/	/	<0.01
	d	0.0021	0	0	0.0044	0.0048	/	/	/	/	/	0.30
	Precise Repair	/	/	/	/	/	0.3012	0	0	0.6537	0.9165	0.20
	Repair-error	/	/	/	/	/	0.4101	0.3402	0.3449	0.4772	0.4897	<0.01
	Repair accuracy	/	/	/	/	/	0.4234	0	0	0.6472	0.7041	0.20
CRTISO (-4bp Processed DSB)	K_{cut}	0.018	0.0116	0.0122	0.0573	0.1156	0.8845	0.5734	0.5879	2.9035	5.832	<0.01
	$K_{processing}$	0.4786	0.2142	0.241	1.0415	2.1503	0.6145	0.281	0.3029	1.3468	2.774	<0.01
	E_{direct}	0.1994	0.0871	0.1063	0.3307	0.3767	0.2561	0.1108	0.1343	0.4295	0.4869	<0.01
	$E_{processed}$	0.0364	0	0	0.0813	0.0962	0.1692	0	0	0.3693	0.4052	0.35
	P_{direct}	0	0	0	1.0673	4.0797	0	0	0	1.3821	5.2705	0.8
	$P_{processed}$	0.0834	0	0	0.2762	0.5968	0.3874	0	0	1.2906	2.7285	0.11
	U	0	0	0	0	0.0004	/	/	/	/	/	0.96
	r	9.3047	2.7449	3.64	665.4331	1676.914	/	/	/	/	/	<0.01
	d	0	0	0	0	0.0002	/	/	/	/	/	0.96
	Precise Repair	/	/	/	/	/	0.3874	0	0	2.394	5.2847	0.1
	Repair-error	/	/	/	/	/	0.4253	0.339	0.3439	0.5089	0.5116	<0.01
	Repair accuracy	/	/	/	/	/	0.4767	0	0	0.8486	0.9252	0.1
PhyB2	K_{cut}	0.0646	0.0432	0.045	0.1171	0.323	0.8257	0.574	0.606	1.7192	8.4772	<0.01
	$K_{processing}$	0.0224	0.0108	0.0125	0.0504	0.0545	0.1244	0.063	0.0706	0.2543	0.2964	<0.01
	E_{direct}	0.0742	0.04	0.0529	0.0886	0.0968	0.4116	0.2176	0.2733	0.4671	0.4857	<0.01
	$E_{processed}$	0.1679	0.0659	0.0872	0.4179	0.6244	0.1206	0.0555	0.0665	0.2502	0.2941	0.01
	P_{direct}	0.0482	0.003	0.0058	0.2327	1.8688	0.2676	0.0175	0.0343	1.128	7.865	0.01
	$P_{processed}$	0	0	0	0	4.00E-04	0	0	0	0	4.00E-04	0.99
	U	0.4049	0	0.3271	0.434	0.4369	/	/	/	/	/	0.03
	r	10.4842	3.449	5.1286	963.6193	6898.781	/	/	/	/	/	<0.01
	d	0	0	0	0.0002	0.007	/	/	/	/	/	0.95
	Precise Repair	/	/	/	/	/	0.2676	0.0175	0.0343	1.128	7.865	0.01
	Repair-error	/	/	/	/	/	0.5323	0.501	0.5053	0.5657	0.5812	<0.01
	Repair accuracy	/	/	/	/	/	0.3346	0.0313	0.0582	0.6659	0.9317	0.01

Table S4. Rates and Flows Estimated by a 4-State Model of DSB Repair

[a]Rates are reported as the number of events per molecule per hour. [b]The flow is reported as the proportion of molecules that experienced that event at the end of the experiment (72h). [c]Confidence intervals are reported as the 1% and 99% percentiles of the estimates obtained from 100 stratified bootstraps of the data, while p.values as the proportion of bootstraps with value smaller or equal than 0 (one-sided test). When none of the 100 bootstraps had value equal to 0 we reported p.values as <0.01. The total proportion of molecules repaired either precisely or generating errors at over the time course are reported as Precise Repair and Repair-error, respectively, while the total proportion of molecules repaired precisely over the total of repaired molecules is reported as Repair accuracy.

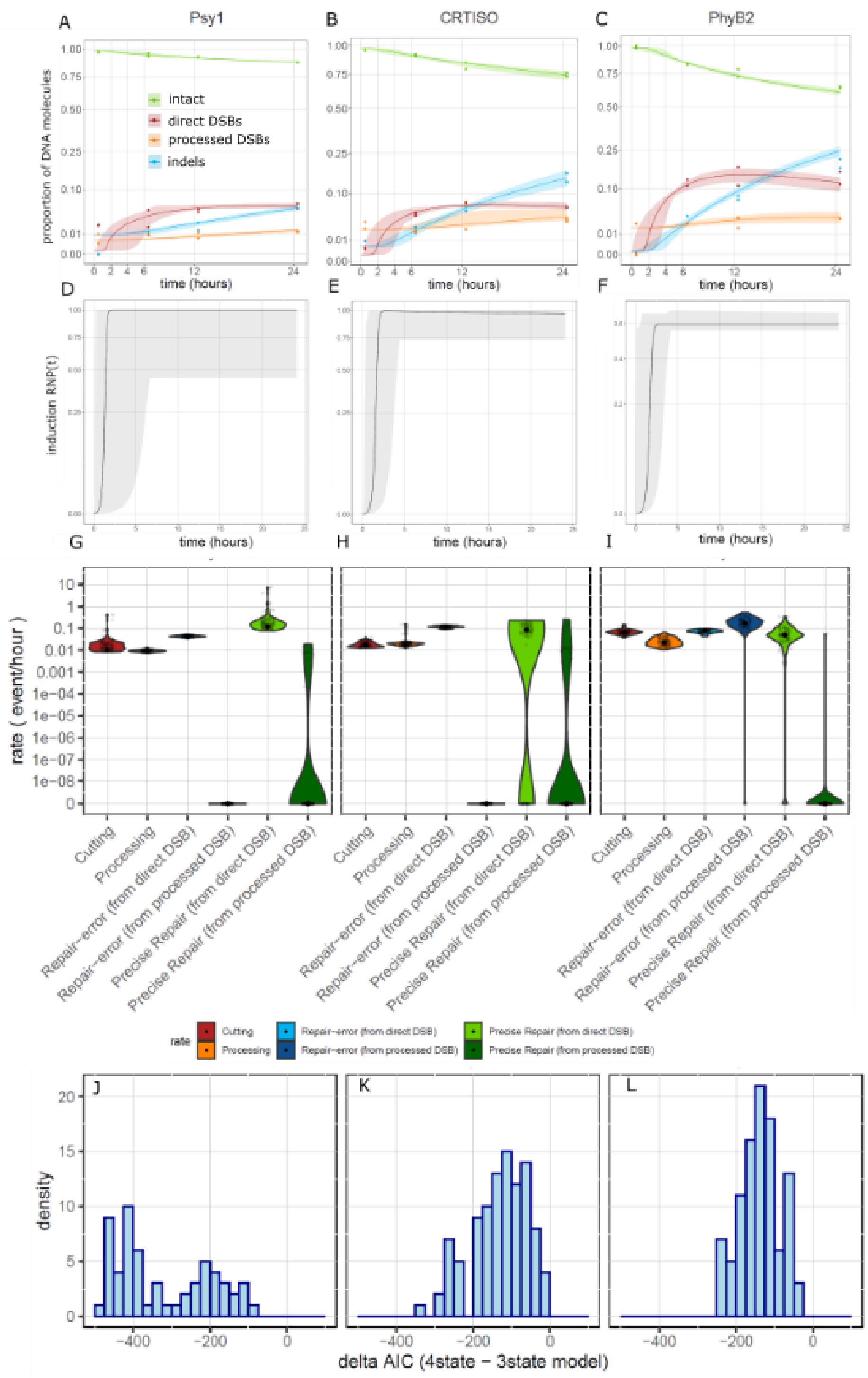

Figure S13. Kinetic model with 4 states and 30 minutes transformation time. A-C) Fit of the 4-state model to the data: A) *Psy1*, B) *CRTISO* and C) PhyB2. Intact molecules are shown in green, DSB in red, indels in blue and processed DSB in orange. Confidence intervals are represented in shadings and calculated from 100 bootstrap samples. D-F) Induction curves for *Psy1* (D), *CRTISO* (E) and PhyB2 (F). The confidence intervals of the induction curves are

indicated in gray shading. G-I) Estimates of the rate constants. The estimate obtained from the original (before bootstrapping) data is shown as a black point, while the distribution of the bootstrap estimates in the violin plot, for *Psy1* (G), *CRTISO* (H) and PhyB2 (I). Transformation time was accounted by setting the moment of transfection with RNPs as time 0h, and the first sampling at 30 minutes after transformation. Support for the 4-state model ia shown as the distribution of differences in AIC between 4 state and 3 state model in bootstraps for Psy1 (J), CRTISO (K) and PhyB2 (L). Negative values show support for the 4-state model.

Target	Process	Rate[a] Constant	CI (bootstrap percentile) [a-c]				Flow[b] at 72h	CI (bootstrap percentile)				
			1%	5%	95%	99%		1%	5%	95%	99%	P.value
Psy1	K_{cut}	0.0104	0.0087	0.009	0.0437	0.3945	0.6374	0.499	0.5414	2.5665	22.1537	<0.01
	$K_{processing}$	0.009	0.0076	0.008	0.0111	0.0132	0.0313	0.0251	0.0289	0.0371	0.0424	<0.01
	E_{direct}	0.0421	0.0337	0.0374	0.0483	0.0496	0.1467	0.1307	0.1352	0.1584	0.1605	<0.01
	$E_{processed}$	0	0	0	0	0	0	0	0	0	0	1
	P_{direct}	0.1181	0.0802	0.0872	0.7188	6.9224	0.4108	0.2737	0.3097	2.3454	21.7813	<0.01
	$P_{processed}$	0	0	0	0.0117	0.0173	0	0	0	0.0114	0.0177	0.81
	U	0	0	0	0.4933	0.519	/	/	/	/	/	0.69
	r	9.9053	2.06	2.5772	46.3207	56.665	/	/	/	/	/	<0.01
	d	0	0	0	0	0	/	/	/	/	/	0.99
	Precise Repair	/	/	/	/	/	0.4108	0.2808	0.316	2.3458	21.7813	<0.01
	Repair-error	/	/	/	/	/	0.1467	0.1307	0.1352	0.1584	0.1605	<0.01
	Repair accuracy	/	/	/	/	/	0.7369	0.6478	0.6701	0.9413	0.9934	<0.01
CRTISO	K_{cut}	0.0179	0.0132	0.0134	0.0282	0.0315	0.8107	0.5475	0.5596	1.1958	1.4586	<0.01
	$K_{processing}$	0.0183	0.0125	0.0143	0.0554	0.147	0.0658	0.0469	0.0527	0.1994	0.5279	<0.01
	E_{direct}	0.1139	0.0937	0.095	0.1295	0.132	0.4082	0.3364	0.3425	0.4734	0.4777	<0.01
	$E_{processed}$	0	0	0	0	0	0	0	0	0	0	1
	P_{direct}	0.0842	0	0	0.1898	0.2022	0.3019	0	0	0.671	0.7041	0.29
	$P_{processed}$	0	0	0	0.073	0.2522	0	0	0	0.1657	0.5061	0.76
	U	0	0	0	0.1943	0.2324	/	/	/	/	/	0.79
	r	8.0722	3.8745	5.1233	21.9117	44.4781	/	/	/	/	/	<0.01
	d	0.002	0	0	0.0042	0.0045	/	/	/	/	/	0.24
	Precise Repair	/	/	/	/	/	0.3019	0	0	0.7107	1.0351	0.21
	Repair-error	/	/	/	/	/	0.4082	0.3364	0.3425	0.4734	0.4777	<0.01
	Repair accuracy	/	/	/	/	/	0.4252	0	0	0.6711	0.7376	0.21
CRTISO (-4bp Processed DSB)	K_{cut}	0.0181	0.011	0.0118	0.0913	0.127	0.8848	0.5606	0.5755	3.9756	6.4488	<0.01
	$K_{processing}$	0.4819	0.1997	0.2122	0.9777	1.3887	0.6152	0.2512	0.2671	1.2921	1.8083	<0.01
	E_{direct}	0.2002	0.0891	0.1029	0.3205	0.3384	0.2557	0.1127	0.131	0.42	0.4254	<0.01
	$E_{processed}$	0.0362	0	0	0.0841	0.0954	0.1672	0	0	0.3785	0.391	0.32
	P_{direct}	0	0	0	1.8278	4.7045	0	0	0	2.3925	5.9277	0.78
	$P_{processed}$	0.0845	0	0	0.2684	0.3953	0.39	0	0	1.2379	1.7572	0.15
	U	0	0	0	0	0.0004	/	/	/	/	/	0.91
	r	6.8712	2.8577	3.2713	646.6363	1001.272	/	/	/	/	/	<0.01
	d	0	0	0	0	1.00E-04	/	/	/	/	/	0.95
	Precise Repair	/	/	/	/	/	0.39	0	0	3.4195	5.9563	0.09
	Repair-error	/	/	/	/	/	0.4228	0.3387	0.347	0.5101	0.5138	<0.01
	Repair accuracy	/	/	/	/	/	0.4798	0	0	0.8745	0.9439	0.09
PhyB2	K_{cut}	0.0645	0.041	0.0464	0.098	0.1452	0.824	0.5733	0.5987	1.3207	2.1339	<0.01
	$K_{processing}$	0.0225	0.0106	0.0124	0.0456	0.0508	0.1243	0.0598	0.0706	0.251	0.2769	<0.01
	E_{direct}	0.0742	0.0433	0.0481	0.0921	0.0967	0.4106	0.236	0.2667	0.4803	0.5035	<0.01
	$E_{processed}$	0.1682	0.0466	0.0661	0.4538	0.4678	0.1205	0.0499	0.0668	0.2481	0.2735	0.01
	P_{direct}	0.0482	0	0.0058	0.1616	0.3337	0.2667	0	0.0338	0.7476	1.5807	0.04
	$P_{processed}$	0	0	0	0	0.0005	0	0	0	0	0.0005	0.99
	U	0.4049	0.3099	0.3658	0.4341	0.4428	/	/	/	/	/	<0.01
	r	7.6111	4.1528	4.6254	23.0124	40.2187	/	/	/	/	/	<0.01
	d	0	0	0	0	0.0017	/	/	/	/	/	0.97
	Precise Repair	/	/	/	/	/	0.2667	0	0.0338	0.7476	1.5807	0.04
	Repair-error	/	/	/	/	/	0.5311	0.4986	0.5036	0.5599	0.5643	<0.01
	Repair accuracy	/	/	/	/	/	0.3343	0	0.0594	0.5773	0.7506	0.04

Table S5. Estimates of the Rates and Induction parameters for the 4-state model of DSB Repair with transformation time for the 72-hour time course.

[a]Rates are reported as the number of events per molecule per hour. [b]The flow is reported as the proportion of molecules that experienced that event at the end of the experiment (72h). [c]Confidence intervals are reported as the 1% and 99% percentiles of the estimates obtained from 100 stratified bootstraps of the data, while p.values as the proportion of bootstraps with value smaller or equal than 0 (one-sided test). When none of the 100 bootstraps had value equal to 0 we reported p.values as <0.01. The total proportion of molecules repaired either precisely or generating errors at over the time course are reported as Precise Repair and Repair-error, respectively, while the total proportion of molecules repaired precisely over the total of repaired molecules is reported as Repair accuracy.

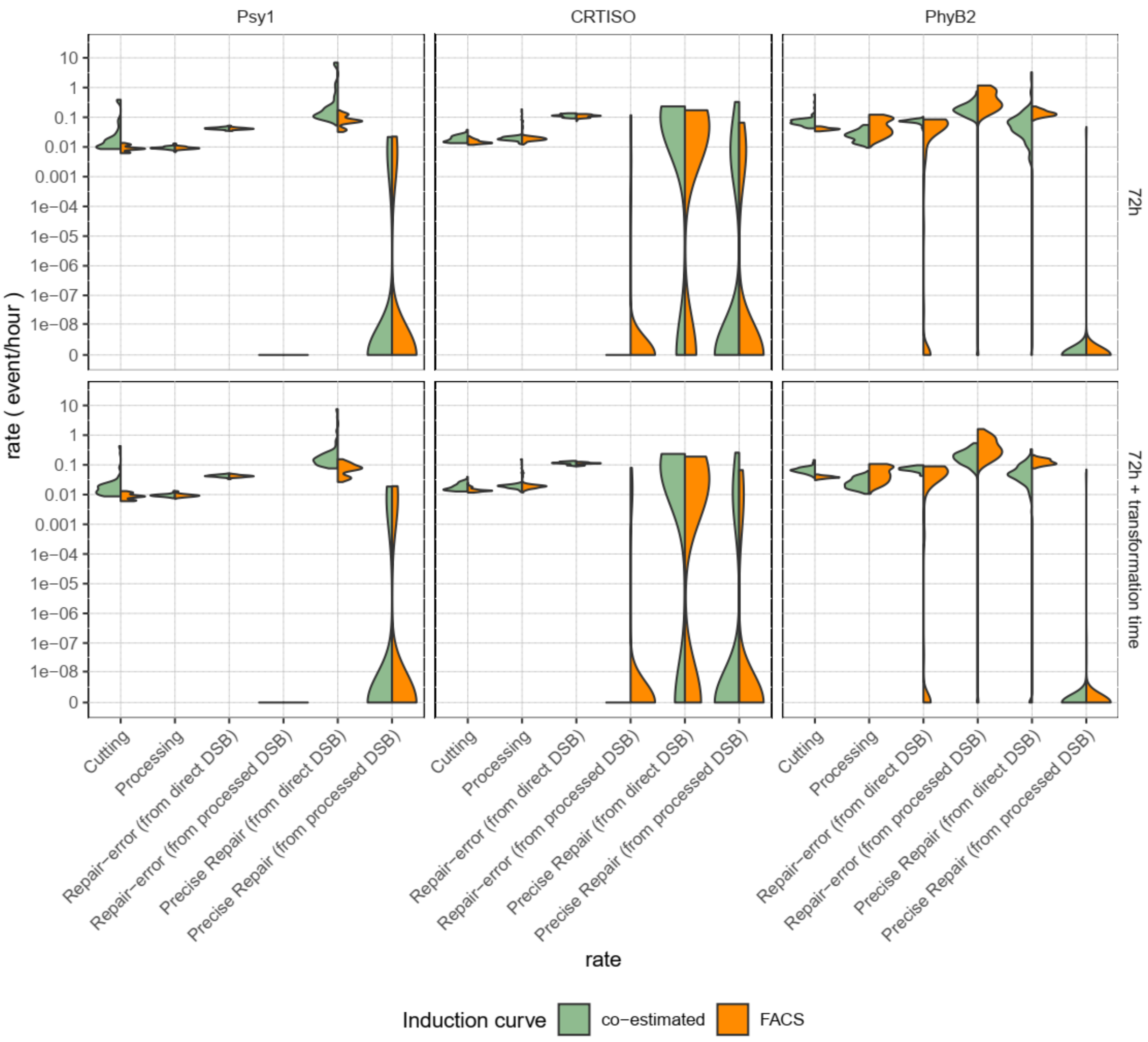

Figure S14. Comparison of estimates when co-estimating induction curve and repair parameters (green) or using the induction curve estimated from FACS data (orange) for the 4 state model in the 72 hour time course. Violin plots represent the bootstrap estimates for the two methods. Note how fixing the induction curve reduces the number of parameters and the uncertainty, especially for PhyB2.

Target	Process	Rate Constant[a]	CI (bootstrap percentile)				Flow at 72h	CI (bootstrap percentile)				
			1%	5%	95%	99%		1%	5%	95%	99%	P.value
Psy1	K_{cut}	0.0088	0.0062	0.0063	0.0125	0.0134	0.511	0.4965	0.4965	0.4965	0.4965	<0.01
	$K_{processing}$	0.0089	0.0079	0.0083	0.0105	0.0115	/	0.0324	0.0324	0.0324	0.0324	<0.01
	E_{direct}	0.0416	0.0351	0.0381	0.045	0.0492	/	0.1481	0.1481	0.1481	0.1481	<0.01
	$E_{processed}$	0	0	0	0	0	/	0	0	0	0	1
	P_{direct}	0.0775	0.0333	0.038	0.1314	0.1484	/	0.27	0.27	0.27	0.27	<0.01
	$P_{processed}$	0	0	0	0.0065	0.0174	/	0	0	0	0	0.89
	U	0.0253	0.0253	0.0253	0.0253	0.0253	/	/	/	/	/	<0.01
	r	85.9775	85.9775	85.9775	85.9775	85.9775	/	/	/	/	/	<0.01
	d	0.0026	0.0026	0.0026	0.0026	0.0026	/	/	/	/	/	<0.01
	Precise Repair	0.0775	0.0333	0.0383	0.1348	0.1484	/	0.27	0.27	0.27	0.27	<0.01
	Repair-error	0.0416	0.0351	0.0381	0.045	0.0492	/	0.1481	0.1481	0.1481	0.1481	<0.01
	Repair accuracy	0.6508	0.4376	0.4673	0.7686	0.7897	/	0.6458	0.6458	0.6458	0.6458	<0.01
CRTISO	K_{cut}	0.0139	0.0118	0.012	0.0201	0.0223	0.6734	0.6046	0.6046	0.6046	0.6046	<0.01
	$K_{processing}$	0.018	0.0131	0.0159	0.0227	0.0508	/	0.0645	0.0645	0.0645	0.0645	<0.01
	E_{direct}	0.1117	0.0906	0.0948	0.1243	0.1267	/	0.4144	0.4144	0.4144	0.4144	<0.01
	$E_{processed}$	0	0	0	0	0.0088	/	0	0	0	0	0.97
	P_{direct}	0.0281	0	0	0.1221	0.158	/	0.0922	0.0922	0.0922	0.0922	0.15
	$P_{processed}$	0	0	0	0.0093	0.0276	/	0	0	0	0	0.88
	U	0.0253	0.0253	0.0253	0.0253	0.0253	/	/	/	/	/	/
	r	85.9775	85.9775	85.9775	85.9775	85.9775	/	/	/	/	/	/
	d	0.0026	0.0026	0.0026	0.0026	0.0026	/	/	/	/	/	/
	Precise Repair	0.0281	0	0	0.1242	0.166	/	0.0922	0.0922	0.0922	0.0922	0.11
	Repair-error	0.1117	0.0927	0.0955	0.1249	0.1301	/	0.4144	0.4144	0.4144	0.4144	<0.01
	Repair accuracy	0.2012	0	0	0.5655	0.6395	/	0.182	0.182	0.182	0.182	0.11
CRTISO (-4bp Processed DSB)	K_{cut}	0.0398	0.0335	0.035	0.048	0.0493	1.4498	1.4974	1.4974	1.4974	1.4974	<0.01
	$K_{processing}$	0.0372	0.0157	0.0187	0.0975	0.1174	/	0.2335	0.2335	0.2335	0.2335	<0.01
	E_{direct}	0.0591	0	0	0.0755	0.0828	/	0.3631	0.3631	0.3631	0.3631	0.09
	$E_{processed}$	0.2906	0.1216	0.1406	0.9641	1.13	/	0.2234	0.2234	0.2234	0.2234	0.01
	P_{direct}	0.1308	0.0883	0.1018	0.1862	0.223	/	0.8654	0.8654	0.8654	0.8654	<0.01
	$P_{processed}$	0	0	0	0	0	/	0	0	0	0	0.99
	U	0.0253	0.0253	0.0253	0.0253	0.0253	/	/	/	/	/	/
	r	85.9775	85.9775	85.9775	85.9775	85.9775	/	/	/	/	/	/
	d	0.0026	0.0026	0.0026	0.0026	0.0026	/	/	/	/	/	/
	Precise Repair	0.1308	0.0883	0.1018	0.1862	0.223	/	0.8654	0.8654	0.8654	0.8654	<0.01
	Repair-error	0.3498	0.1955	0.2174	0.9642	1.1336	/	0.5865	0.5865	0.5865	0.5865	<0.01
	Repair accuracy	0.2722	0.1044	0.1306	0.4021	0.4121	/	0.5961	0.5961	0.5961	0.5961	<0.01
PhyB2	K_{cut}	0.0088	0.0062	0.0063	0.0125	0.0134	0.511	0.4965	0.4965	0.4965	0.4965	<0.01
	$K_{processing}$	0.0089	0.0079	0.0083	0.0105	0.0115	/	0.0324	0.0324	0.0324	0.0324	<0.01
	E_{direct}	0.0416	0.0351	0.0381	0.045	0.0492	/	0.1481	0.1481	0.1481	0.1481	<0.01
	$E_{processed}$	0	0	0	0	0	/	0	0	0	0	1
	P_{direct}	0.0775	0.0333	0.038	0.1314	0.1484	/	0.27	0.27	0.27	0.27	<0.01
	$P_{processed}$	0	0	0	0.0065	0.0174	/	0	0	0	0	0.90
	U	0.0253	0.0253	0.0253	0.0253	0.0253	/	/	/	/	/	/
	r	85.9775	85.9775	85.9775	85.9775	85.9775	/	/	/	/	/	/
	d	0.0026	0.0026	0.0026	0.0026	0.0026	/	/	/	/	/	/
	Precise Repair	0.0775	0.0333	0.0383	0.1348	0.1484	/	0.27	0.27	0.27	0.27	<0.01
	Repair-error	0.0416	0.0351	0.0381	0.045	0.0492	/	0.1481	0.1481	0.1481	0.1481	<0.01
	Repair accuracy	0.6508	0.4376	0.4673	0.7686	0.7897	/	0.6458	0.6458	0.6458	0.6458	<0.01

Table S6. Estimates of the repair rates for the 4-state model of DSB Repair with induction parameters fixed to those estimated from FACS data.

[a]Rates are reported as the number of events per molecule per hour. [b]The flow is reported as the proportion of molecules that experienced that event at the end of the experiment (72h). [c]Confidence intervals are reported as the 1% and 99% percentiles of the estimates obtained from 100 stratified bootstraps of the data, while p.values as the proportion of bootstraps with value smaller or equal than 0 (one-sided test). When none of the 100 bootstraps had value equal to 0 we reported p.values as <0.01. The total proportion of molecules repaired either precisely or generating errors at over the time course are reported as Precise Repair and Repair-error, respectively, while the total proportion of molecules repaired precisely over the total of repaired molecules is reported as Repair accuracy.

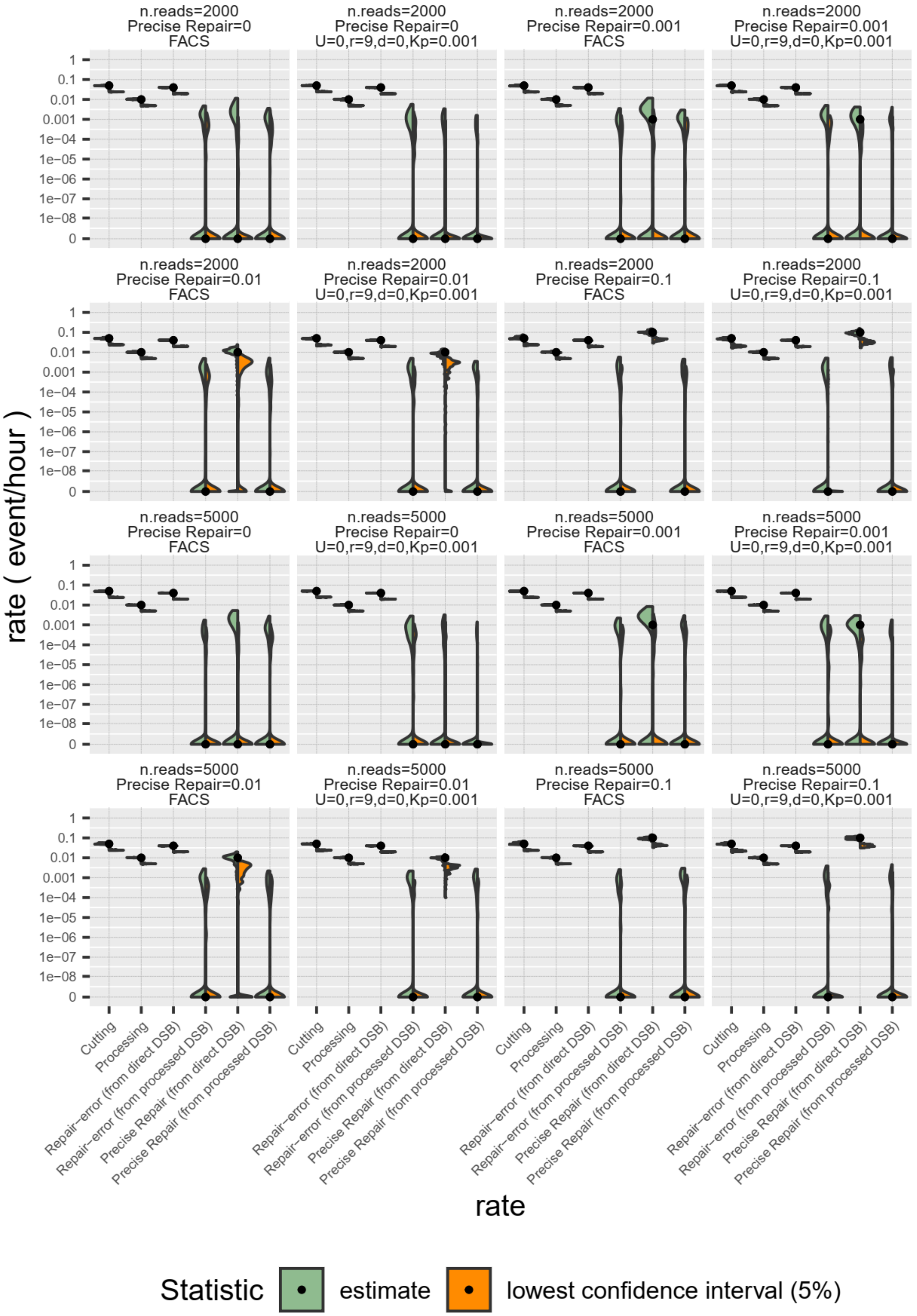
n.reads=2000
Precise Repair=0
FACS
n.reads=2000
Precise Repair=0
U=0,r=9,d=0,Kp=0.001
n.reads=2000
Precise Repair=0.001
FACS
n.reads=2000
Precise Repair=0.001
U=0,r=9,d=0,Kp=0.001
n.reads=2000
Precise Repair=0.01
FACS
n.reads=2000
Precise Repair=0.01
U=0,r=9,d=0,Kp=0.001
n.reads=2000
Precise Repair=0.1
FACS
n.reads=2000
Precise Repair=0.1
U=0,r=9,d=0,Kp=0.001
n.reads=5000
Precise Repair=0
FACS
n.reads=5000
Precise Repair=0
U=0,r=9,d=0,Kp=0.001
n.reads=5000
Precise Repair=0.001
FACS
n.reads=5000
Precise Repair=0.001
U=0,r=9,d=0,Kp=0.001
n.reads=5000
Precise Repair=0.01
FACS
n.reads=5000
Precise Repair=0.01
U=0,r=9,d=0,Kp=0.001
n.reads=5000
Precise Repair=0.1
FACS
n.reads=5000
Precise Repair=0.1
U=0,r=9,d=0,Kp=0.001
rate (event/hour)
1
0.1
0.01
0.001
1e−04
1e−05
1e−06
1e−07
1e−08
0
Cutting
Processing
Repair−error (from direct DSB)
Repair−error (from processed DSB)
Precise Repair (from direct DSB)
Precise Repair (from processed DSB)
rate
Statistic
estimate
lowest confidence interval (5%)

Figure S15. Simulations and testing of the 4 state model. Violin plots showing the distributions of the point estimates (green, left violin) and lowest confidence intervals (orange, right violin) of rate parameters for 100 simulations. Simulated values shown as a black dot. Different values of precise repair (0,0.1,0.01 or 0.001 events/hour) and numbers of reads (2000 and 5000) were simulated. Two sets of representative induction curve parameters shown: a curve with no decay (r=9 U=0 and d=0) and a curve with very rapid induction and a slow decay (U=0.02529, r=85.9774, d=0.00263), which was estimated from the FACS as described in the text and shown in Fig. S9. For all conditions, $K_{processing}$ (indicated as Kp) was set to 0.001. Simulated parameters were generally selected to reflect those observed in our data. Note that across all conditions, very little false positives are observed when precise repair is absent, i.e. the orange distribution is centered around 0 when Precise Repair=0; and for those, the point estimate is lower than 0.01.

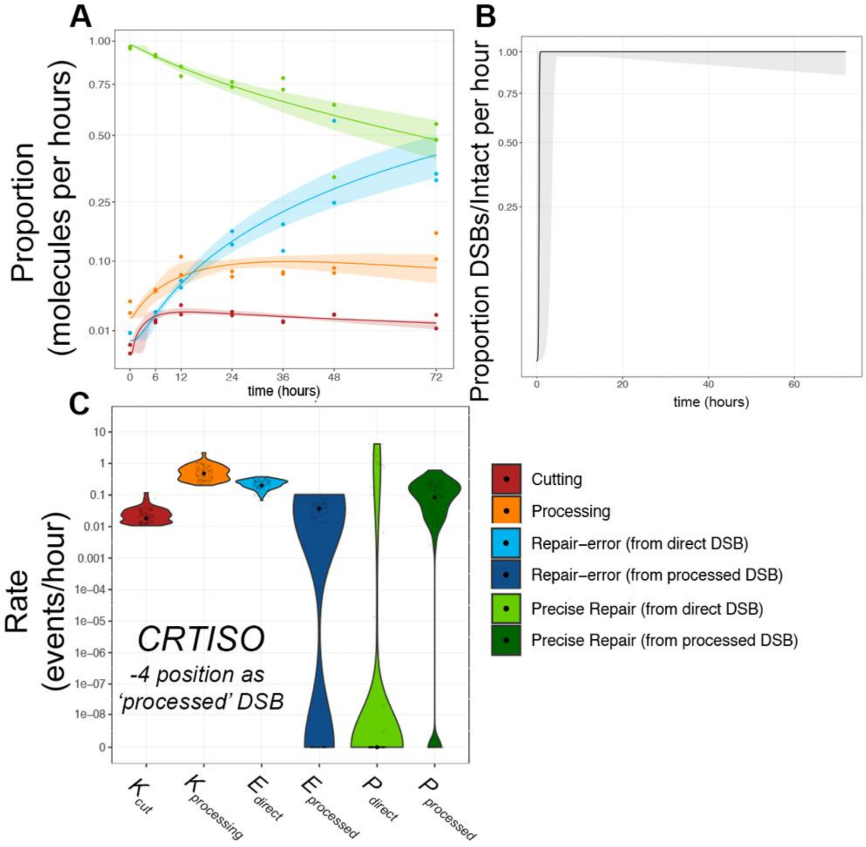

Figures S16. Complex Dynamics at CRTISO A) Fit of the model to the data for CRTISO when the -4 bp positioned DSB is considered as 'processed' with Confidence Intervals (CIs) represented in shading and calculated from 100 iterations of the bootstrapping. B) Induction curve with CIs from the stratified bootstrap indicated in gray shading. C) Violin plots of rate constants estimated in terms of proportion per hour, smoothed distribution of the estimates obtained through the bootstrap procedure. The estimate obtained from the original (before bootstrapping) data estimate of 100 iterations of bootstrapping is shown as a black point.

Target	Process	Rate[a] (proportion of total event/hour)	CI [1-99%][a,c]	Flow[b] after 24h (proportion of total molecules)	CI [1-99%][b,c]	P.value
Psy1	Cutting	0.0164	0.0145-0.026	0.34	0.2772-0.4004	<0.01
	Repair-error	0.0777	0.0684-0.0925	0.1328	0.1159-0.1544	<0.01
	Precise Repair	0.069	0.0149-0.1143	0.1179	0.0271-0.1925	<0.01
	e (processed DSB)	0.1228	0.1025-0.1457	/	/	<0.01
	U (uncut fraction)	0	0-0.2736	/	/	0.98
	r (induction speed)	47.8246	36.8609-338.6245	/	/	<0.01
	d (induction decay)	0	0-1e-04	/	/	<0.01
	Repair accuracy	0.4703	0.1521-0.6203	0.4703	0.1521-0.6203	<0.01
CRTISO	Cutting	0.0251	0.021-0.0297	0.4268	0.3761-0.4663	<0.01
	Repair-error	0.0538	0.0449-0.086	0.1906	0.1712-0.2348	<0.01
	Precise Repair	0	0-0.0201	0	0-0.0604	0.93
	e (processed DSB)	0.1016	0.0734-0.1144	/	/	<0.01
	U (uncut fraction)	0	0-0.0017	/	/	0.99
	r (induction speed)	7.4007	6.5972-9.094	/	/	<0.01
	d (induction decay)	0	0-0.0191	/	/	<0.01
	Repair accuracy	0	0-0.2547	0	0-0.2547	0.93
PhyB2	Cutting	0.0643	0.056-10	0.4107	0.162-183.8372	<0.01
	Repair-error	0.0274	0.0183-0.0327	0.1192	0.0527-0.1537	<0.01
	Precise Repair	0.0365	0.0014-52.529	0.1585	0.0046-174.3338	0.01
	e (processed DSB)	0.1455	0.1197-0.1619	/	/	<0.01
	U (uncut fraction)	0	0-0.7511	/	/	0.8
	r (induction speed)	37.4423	0-224.7432	/	/	0.03
	d (induction decay)	0.0904	0-0.8167	/	/	<0.01
	Repair accuracy	0.5708	0.0553-0.9997	0.5708	0.0553-0.9996	0.01

Table S7. Estimates of the Rates and Induction parameters for the 3-state model of DSB Repair in the high-resolution time course with transformation time for the 24-hour time course.

[a]Rates are reported as the number of events per molecule per hour. [b]The flow is reported as the proportion of molecules that experienced the specific event at the end of the experiment. [c]Confidence intervals (CI) are reported as the 1% and 99% percentiles of the estimates obtained from 100 stratified bootstraps of the data, while p.values as the proportion of bootstraps with value smaller or equal than 0 (one-sided test). When none of the 100 bootstraps had value equal to 0 we reported p.values as <0.01. The induction curve is modeled as a logistic increase in activity of the RNPs with speed *r*, a fraction *U* of cells upon which the RNPs do not cut DNA, and a decay *d*. An error rate *e* describes the proportion of DSBs which show DSB ends not coinciding with the expected position (see *Determining the parameters of the induction curve).* Repair accuracy is computed as the proportion of DSBs repaired precisely over all repaired DSBs at the end of the time course (24h). Transformation time was accounted for by setting time 0h to the moment of transfection with RNPs and the first sampling at 30 minutes after transfection.

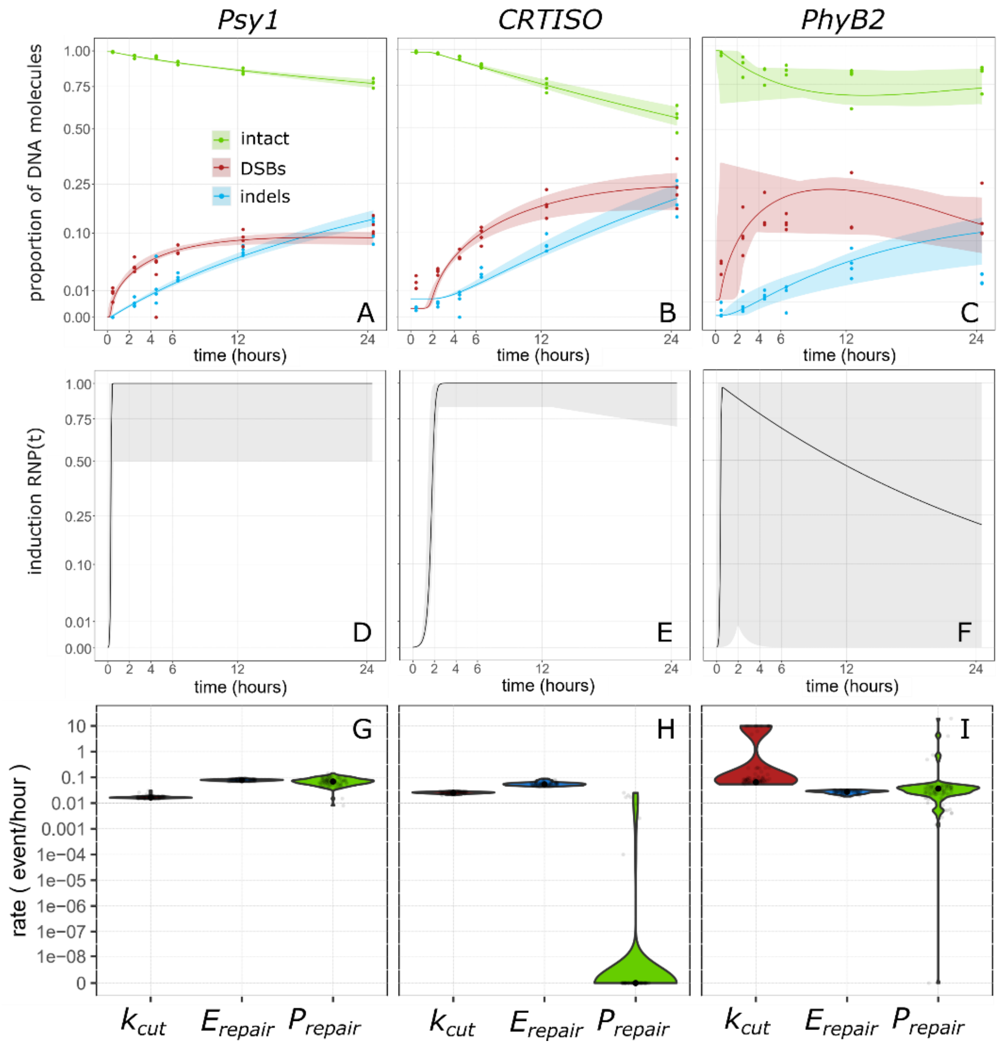

Figure S17. **The 3-state model of DSB induction and repair for the 24h high resolution time course with 30-minute transformation time.** A-C) Fit of the 4-state model to the data for (A) *Psy1*, (B) *CRTISO* and (C) PhyB2. Intact molecules are shown in green, DSB in red and indels in blue. Shadings represent uncertainty computed as the range of 100 iterations of time-stratified bootstraps. D-F) Induction curves for (D) *Psy1*, (E) *CRTISO* and (F) *PhyB2*. The confidence intervals for the induction curves are indicated in gray shading. G-I) Violin plots of rate constants estimated in terms of proportion per hour, smoothed distribution of the estimates obtained through the bootstrap procedure for *Psy1* (G), *CRTISO* (H) and PhyB2 (I). The estimate obtained from the original (before bootstrapping) data is shown as a black point. Transformation time was accounted for by setting time 0h to the moment of transfection with RNPs and the first sampling at 30 minutes after transfection.

Target	Process	Rate[a] Constant	CI (bootstrap percentile)[c]				Flow[b] at 24h	CI (bootstrap percentile)[c]				P.value
			1%	5%	95%	99%		1%	5%	95%	99%	
	K_{cut}	0.0153	0.0129	0.0135	0.0185	0.0213	0.3199	0.2605	0.2775	0.3607	0.3929	<0.01
	$K_{processing}$	0.0258	0.0164	0.0188	0.0393	0.0496	0.0384	0.0243	0.0283	0.0598	0.0721	<0.01
	E_{direct}	0.0886	0.075	0.079	0.101	0.106	0.132	0.1164	0.1209	0.1472	0.1528	<0.01
	$E_{processed}$	0	0	0	0	0	0	0	0	0	0	1
	P_{direct}	0.0506	0	0.0019	0.0907	0.106	0.0754	0	0.003	0.1317	0.1573	0.04
	$P_{processed}$	0.0666	0	0.0094	0.1681	0.2128	0.0173	0	0.0028	0.0427	0.0555	0.02
	U	0	0	0	0	0.3585	/	/	/	/	/	0.96
Psy1	r	14191.96	108.6746	3022.882	16996.13	35528.11	/	/	/	/	/	<0.01
	d	0	0	0	0	0.0071	/	/	/	/	/	0.97
	Precise Repair	0.1171	0.0525	0.0679	0.2086	0.2433	0.0927	0.0269	0.0378	0.1449	0.1771	<0.01
	Repair-error	0.0886	0.075	0.079	0.101	0.106	0.132	0.1164	0.1209	0.1472	0.1528	<0.01
	Repair accuracy	0.5694	0.3923	0.4388	0.6845	0.7252	0.4125	0.1735	0.2129	0.5403	0.5875	<0.01
	K_{cut}	0.025	0.0211	0.022	0.0281	0.0285	0.4308	0.381	0.3926	0.4714	0.4822	<0.01
	$K_{processing}$	0.011	0.0097	0.01	0.0292	0.0362	0.0361	0.0288	0.0319	0.0897	0.1066	<0.01
	E_{direct}	0.0587	0.0487	0.0507	0.0771	0.0837	0.1927	0.174	0.1748	0.211	0.2265	<0.01
	$E_{processed}$	0	0	0	0.063	0.1071	0	0	0	0.0202	0.0467	0.89
	P_{direct}	0	0	0	0	0	0	0	0	0	0	1
	$P_{processed}$	0	0	0	0.1593	0.2547	0	0	0	0.0491	0.083	0.65
CRTISO	U	0	0	0	0	0	/	/	/	/	/	1
	r	9.6577	7.9924	8.6778	10.621	11.0425	/	/	/	/	/	<0.01
	d	0	0	0	0	0.0033	/	/	/	/	/	0.98
	Precise Repair	0	0	0	0.1593	0.2547	0	0	0	0.0491	0.083	0.65
	Repair-error	0.0587	0.0487	0.0507	0.1366	0.1738	0.1927	0.174	0.1748	0.2297	0.2455	<0.01
	Repair accuracy	0	0	0	0.7101	0.8012	0	0	0	0.2036	0.3085	0.65
	K_{cut}	0.0568	0.0496	0.0516	8.4289	9.9997	0.4274	0.3159	0.3242	47.0672	145.2906	<0.01
	$K_{processing}$	0.0577	0.0399	0.0412	3.1407	7.4791	0.2112	0.0985	0.1408	8.0928	19.1765	<0.01
	E_{direct}	0.0316	0.0218	0.0233	0.037	0.0383	0.1157	0.0559	0.06	0.144	0.1502	<0.01
	$E_{processed}$	0	0	0	0	0	0	0	0	0	0	0.94
	P_{direct}	0	0	0	12.4147	48.9995	0	0	0	29.6013	137.0459	0.79
	$P_{processed}$	0.2514	0.1193	0.1427	20.2908	48.4447	0.1812	0.0716	0.1007	7.9462	18.4001	<0.01
	U	0	0	0	0.8042	0.8267	/	/	/	/	/	0.71
PhyB2	r	7632.608	6.7828	6.8973	11995.22	18632.53	/	/	/	/	/	<0.01
	d	0.0798	0	0	0.0939	0.0949	/	/	/	/	/	0.11
	Precise Repair	0.2514	0.1427	0.1688	44.5517	59.208	0.1812	0.1068	0.119	45.7337	137.0845	<0.01
	Repair-error	0.0316	0.0218	0.0233	0.037	0.0383	0.1157	0.0559	0.06	0.144	0.1502	0
	Repair accuracy	0.8884	0.8064	0.8235	0.9995	0.9996	0.6105	0.46	0.4819	0.9985	0.9995	0

Table S8. Estimates of the Rates and Induction parameters for the 4-state model of DSB Repair in the high-resolution 24-hour time course.

[a]Rates are reported as the number of events per molecule per hour. [b]The flow is reported as the proportion of molecules that experienced the specific event at the end of the experiment. [c]Confidence intervals (CI) are reported as the 1% and 99% percentiles of the estimates obtained from 100 stratified bootstraps of the data, while p.values as the proportion of bootstraps with value smaller or equal than 0 (one-sided test). When none of the 100 bootstraps had value equal to 0 we reported p.values as <0.01. The induction curve is modeled as a logistic increase in activity of the RNPs with speed r, a fraction U of cells upon which the RNPs do not cut DNA, and a decay d. An error rate e describes the proportion of DSBs which show DSB ends not coinciding with the expected position (see *Determining the parameters of the induction curve).* Repair accuracy is computed as the proportion of DSBs repaired precisely over all repaired DSBs at the end of the time course (24h).

Target	Process	Rate[a] Constant	CI (bootstrap percentile[c])				Flow[b] at 24h	CI (bootstrap percentile)[c]				P.value
			1%	5%	95%	99%		1%	5%	95%	99%	
Psy1	K_{cut}	0.0153	0.0136	0.0138	0.0183	0.0226	0.3164	0.2757	0.2849	0.36	0.3871	<0.01
	$K_{processing}$	0.0258	0.0177	0.0191	0.0377	0.0408	0.0378	0.0255	0.0277	0.057	0.0619	<0.01
	E_{direct}	0.0886	0.0784	0.081	0.0992	0.1043	0.1301	0.1139	0.1179	0.1481	0.1519	<0.01
	$E_{processed}$	0	0	0	0	0	0	0	0	0	0	1
	P_{direct}	0.0505	0	0.006	0.1025	0.114	0.0742	0	0.0098	0.1413	0.1627	0.05
	$P_{processed}$	0.0665	0	0.0173	0.1651	0.1886	0.0169	0	0.004	0.04	0.0454	0.02
	U	0	0	0	0	0.2245	/	/	/	/	/	0.96
	r	47.7409	23.3236	36.9157	49.0101	67.3469	/	/	/	/	/	0
	d	0	0	0	0	0.0006	/	/	/	/	/	0.98
	Precise Repair	/	/	/	/	/	0.0911	0.031	0.0453	0.1466	0.1738	<0.01
	Repair-error	/	/	/	/	/	0.1301	0.1139	0.1179	0.1481	0.1519	<0.01
	Repair accuracy	/	/	/	/	/	0.4118	0.1714	0.2364	0.5432	0.5888	<0.01
CRTISO	K_{cut}	0.025	0.0216	0.0221	0.0283	0.0289	0.4237	0.3806	0.3908	0.4633	0.4762	<0.01
	$K_{processing}$	0.011	0.0091	0.0098	0.0317	0.0402	0.035	0.0249	0.0303	0.0942	0.1134	<0.01
	E_{direct}	0.0587	0.0491	0.0508	0.0802	0.0906	0.1868	0.1688	0.1715	0.2081	0.2188	<0.01
	$E_{processed}$	0	0	0	0.0139	0.0961	0	0	0	0.0046	0.0356	0.91
	P_{direct}	0	0	0	0	0	0	0	0	0	0	1
	$P_{processed}$	0	0	0	0.1809	0.2788	0	0	0	0.0649	0.09	0.6
	U	0	0	0	0	0	/	/	/	/	/	1
	r	7.148	6.3964	6.6005	7.8038	8.3785	/	/	/	/	/	<0.01
	d	0	0	0	0	0	/	/	/	/	/	0.99
	Precise Repair	/	/	/	/	/	0	0	0	0.0649	0.09	0.6
	Repair-error	/	/	/	/	/	0.1868	0.169	0.172	0.2143	0.2352	<0.01
	Repair accuracy	/	/	/	/	/	0	0	0	0.2476	0.3379	0.6
PhyB2	K_{cut}	0.0584	0.0516	0.0545	9.9042	9.9978	0.4291	0.2665	0.3064	70.3205	153.3423	<0.01
	$K_{processing}$	0.0574	0.0408	0.044	4.8661	8.3813	0.2095	0.1096	0.15	10.8505	18.0534	<0.01
	E_{direct}	0.0316	0.0209	0.0226	0.0369	0.0377	0.1153	0.0527	0.0554	0.1424	0.1495	<0.01
	$E_{processed}$	0	0	0	0	0	0	0	0	0	0	0.9697
	P_{direct}	0	0	0	23.1448	63.5755	0	0	0	53.7007	140.2806	0.8081
	$P_{processed}$	0.2498	0.14	0.1701	34.0406	49.4896	0.1786	0.0872	0.1126	10.5255	17.3475	0
	U	0	0	0	0.8224	0.8296	/	/	/	/	/	0.69
	r	37.5554	5.2684	5.4093	204.3838	464.7601	/	/	/	/	/	<0.01
	d	0.0782	0	0	0.0905	0.0949	/	/	/	/	/	0.1
	Precise Repair	/	/	/	/	/	0.1786	0.1096	0.1185	68.2015	145.6231	<0.01
	Repair-error	/	/	/	/	/	0.1153	0.0527	0.0554	0.1424	0.1495	<0.01
	Repair accuracy	/	/	/	/	/	0.6077	0.4796	0.4898	0.9991	0.9996	<0.01

Table S9. Estimates of the Rates and Induction parameters for the 4-state model of DSB Repair in the high-resolution 24-hour time course with transformation time.

[a]Rates are reported as the number of events per molecule per hour. [b]The flow is reported as the proportion of molecules that experienced the specific event at the end of the experiment. [c]Confidence intervals (CI) are reported as the 1% and 99% percentiles of the estimates obtained from 100 stratified bootstraps of the data, while p.values as the proportion of bootstraps with value smaller or equal than 0 (one-sided test). When none of the 100 bootstraps had value equal to 0 we reported p.values as <0.01. The induction curve is modeled as a logistic increase in activity of the RNPs with speed *r*, a fraction *U* of cells upon which the RNPs do not cut DNA, and a decay *d*. An error rate *e* describes the proportion of DSBs which show DSB ends not coinciding with the expected position (see *Determining the parameters of the induction curve).* Repair accuracy is computed as the proportion of DSBs repaired precisely over all repaired DSBs at the end of the time course (24h). Transformation time was accounted for by setting time 0h to the moment of transfection with RNPs and the first sampling at 30 minutes after transfection.

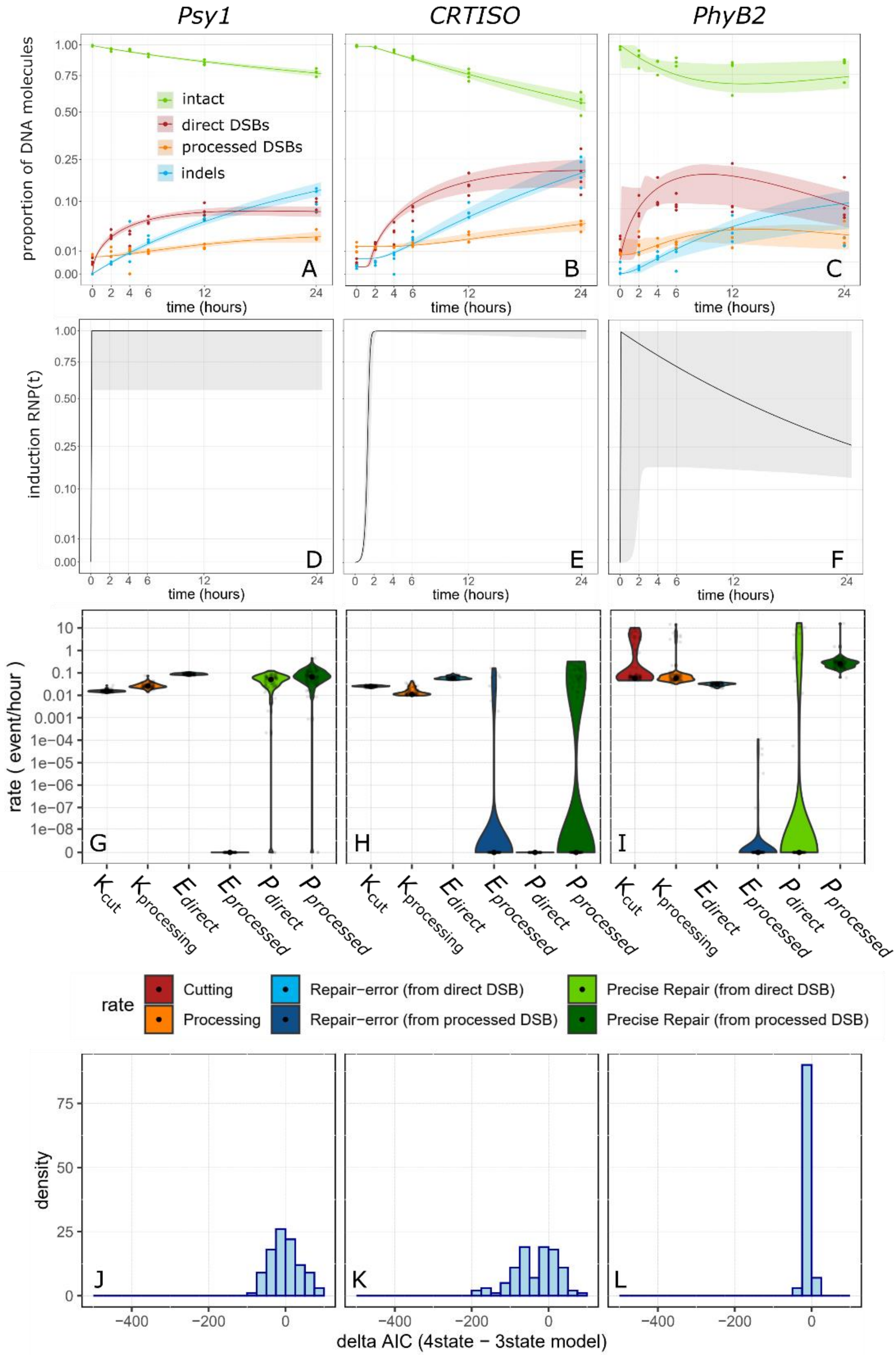

Psy1
CRTISO
PhyB2
proportion of DNA molecules
intact
direct DSBs
processed DSBs
indels
A
B
C
time (hours)
induction RNP(t)
D
E
F
rate (event/hour)
G
H
I
K cut
K processing
E direct
E processed
P direct
P processed
rate
Cutting
Processing
Repair−error (from direct DSB)
Repair−error (from processed DSB)
Precise Repair (from direct DSB)
Precise Repair (from processed DSB)
density
J
K
L
delta AIC (4state − 3state model)

Figure S18. **The 4-state model of DSB induction and repair for the 24h high resolution time course.** Figures are relative to Psy1 (left), CRTISO (middle) and PhyB2 (right). A-C) Fit of the model to the data for *Psy1* (A) and *CRTISO* (B) and PhyB2 (C) and their respective induction curves (D-F). Shadings represent uncertainty computed as the range of 100 iterations of time-stratified boostrap. G-I) Estimates for the rates. Light grey dots indicate individual bootstrap iterations. J-L) Histogram of the differences in AIC (Delta AIC, where AIC stands for Akaike Information Criterion) between the 4-state model and the 3-state model for *Psy1* (J), *CRTISO* (K) and PhyB2 (L) obtained for each iteration of the bootstrapped data. AIC takes into account the likelihood of the models as well as its complexity, i.e. the number of parameters in the model, to establish which model is best supported by the data. Delta AIC values higher than 0 indicate higher support for the simpler model (3-state model), while negative values support the more complex model (4-state model). A Delta AIC of ~-6 corresponds to a relative likelihood of 0.05, indicating strong support for the more complex model.

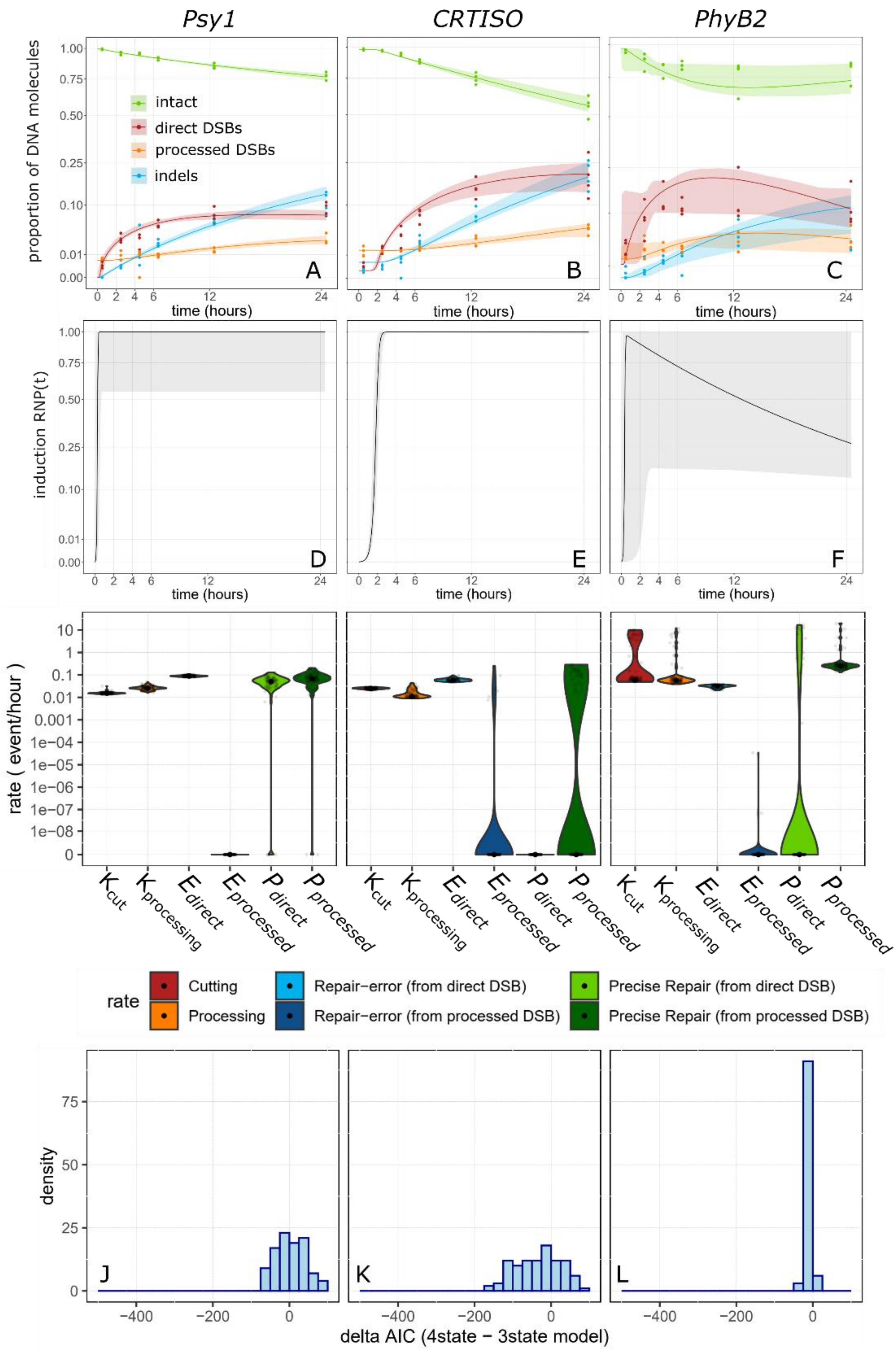

Psy1
CRTISO
PhyB2
proportion of DNA molecules
intact
direct DSBs
processed DSBs
indels
A
B
C
time (hours)
induction RNP(t)
D
E
F
rate (event/hour)
K cut
K processing
E direct
E processed
P direct
P processed
rate
Cutting
Processing
Repair−error (from direct DSB)
Repair−error (from processed DSB)
Precise Repair (from direct DSB)
Precise Repair (from processed DSB)
density
J
K
L
delta AIC (4state − 3state model)

Figure S19. **The 4-state model of DSB induction and repair for the 24h high resolution time course with transformation time.** Figures are relative to Psy1 (left), CRTISO (middle) and PhyB2 (right). A-C) Fit of the model to the data for *Psy1* (A) and *CRTISO* (B) and PhyB2 (C) and their respective induction curves (D-F). Shadings represent uncertainty computed as the range of 100 iterations of time-stratified boostrap. G-I) Estimates for the rates. Light grey dots indicate individual bootstrap iterations. J-L) Histogram of the differences in AIC (Delta AIC, where AIC stands for Akaike Information Criterion) between the 4-state model and the 3-state model for *Psy1* (J), *CRTISO* (K) and PhyB2 (L) obtained for each iteration of the bootstrapped data. AIC takes into account the likelihood of the models as well as its complexity, i.e. the number of parameters in the model, to establish which model is best supported by the data. Delta AIC values higher than 0 indicate higher support for the simpler model (3-state model), while negative values support the more complex model (4-state model). A Delta AIC of ~-6 corresponds to a relative likelihood of 0.05, indicating strong support for the more complex model. Transformation time was accounted for by setting time 0h to the moment of transfection with RNPs and the first sampling at 30 minutes after transfection.

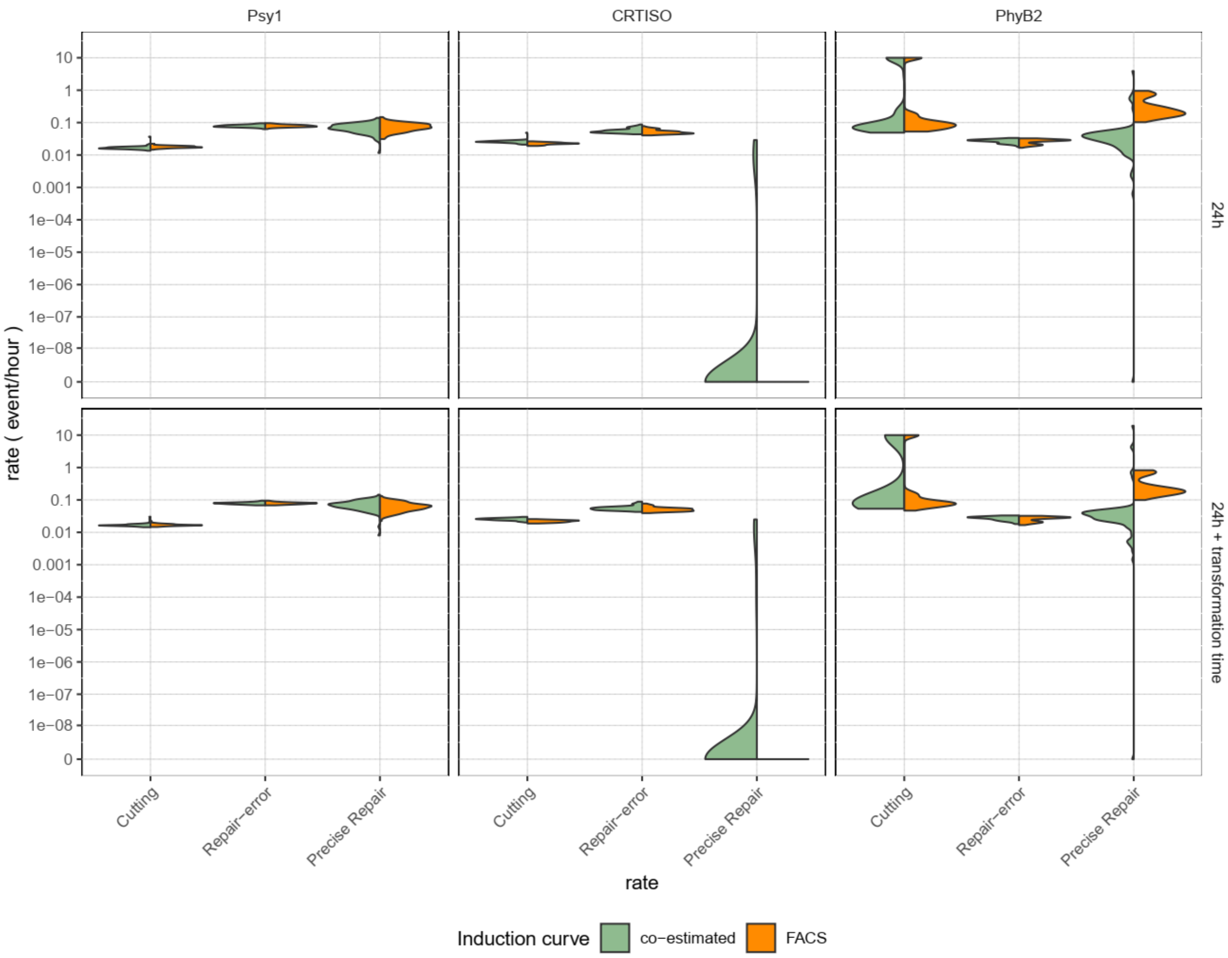

Figure S20. Comparison of estimates when co-estimating induction curve and repair parameters (green) or using the induction curve estimated from FACS data (orange) for the 3-state model in the 24 hour time course. Violin plots represent the bootstrap estimates for the two methods.

Target	Process	Rate[a] (proportion of total event/hour)	CI [1-99%][a,c]	Flow[b] after 24h (proportion of total molecules)	CI [1-99%] [b,c]	P.value
Psy1	Cutting	0.0177	0.015-0.022	0.3499	0.3543-0.3543	<0.01
	Repair-error	0.078	0.0673-0.093	/	0.1313-0.1313	<0.01
	Precise Repair	0.0729	0.035-0.1193	/	0.143-0.143	<0.01
	Error	0.1224	0.1008-0.1418	/	/	<0.01
	U (uncut fraction)	0.0253	0.0253-0.0253	/	/	/
	r (induction speed)	85.9775	85.9775-85.9775	/	/	/
	d (induction decay)	0.0026	0.0026-0.0026	/	/	/
	Repair accuracy	0.4831	0.305-0.6075	/	0.5214-0.5214	<0.01
CRTISO	Cutting	0.0227	0.0193-0.0256	0.3959	0.4174-0.4174	<0.01
	Repair-error	0.0495	0.0418-0.0703	/	0.1731-0.1731	<0.01
	Precise Repair	0	0-0	/	0-0	1
	Error	0.1043	0.0806-0.1165	/	/	<0.01
	U (uncut fraction)	0.0253	0.0253-0.0253	/	/	/
	r (induction speed)	85.9775	85.9775-85.9775	/	/	/
	d (induction decay)	0.0026	0.0026-0.0026	/	/	/
	Repair accuracy	0	0-0	/	0-0	1
PhyB2	Cutting	0.0832	0.0535-10	25.3018	1.1783-1.1783	<0.01
	Repair-error	0.0276	0.0181-0.0321	/	0.152-0.152	<0.01
	Precise Repair	0.2376	0.115-54.9736	/	0.7973-0.7973	<0.01
	Error	0.1464	0.1262-0.1669	/	/	<0.01
	U (uncut fraction)	0.0253	0.0253-0.0253	/	/	/
	r (induction speed)	85.9775	85.9775-85.9775	/	/	/
	d (induction decay)	0.0026	0.0026-0.0026	/	/	/
	Repair accuracy	0.896	0.7847-0.9997	/	0.8399-0.8399	<0.01

Table S10. Estimates of the Rates and Induction parameters for the 3-state model of DSB Repair for the 24-hour time course, with induction parameters fixed to those estimated from FACS data (U=0.0253, r=85.9775, d=0.0026, Fig. S9).

[a]Rates are reported as the number of events per molecule per hour. [b]The flow is reported as the proportion of molecules that experienced the specific event at the end of the experiment. [c]Confidence intervals (CI) are reported as the 1% and 99% percentiles of the estimates obtained from 100 stratified bootstraps of the data, while p.values as the proportion of bootstraps with value smaller or equal than 0 (one-sided test). When none of the 100 bootstraps had value equal to 0 we reported p.values as <0.01.

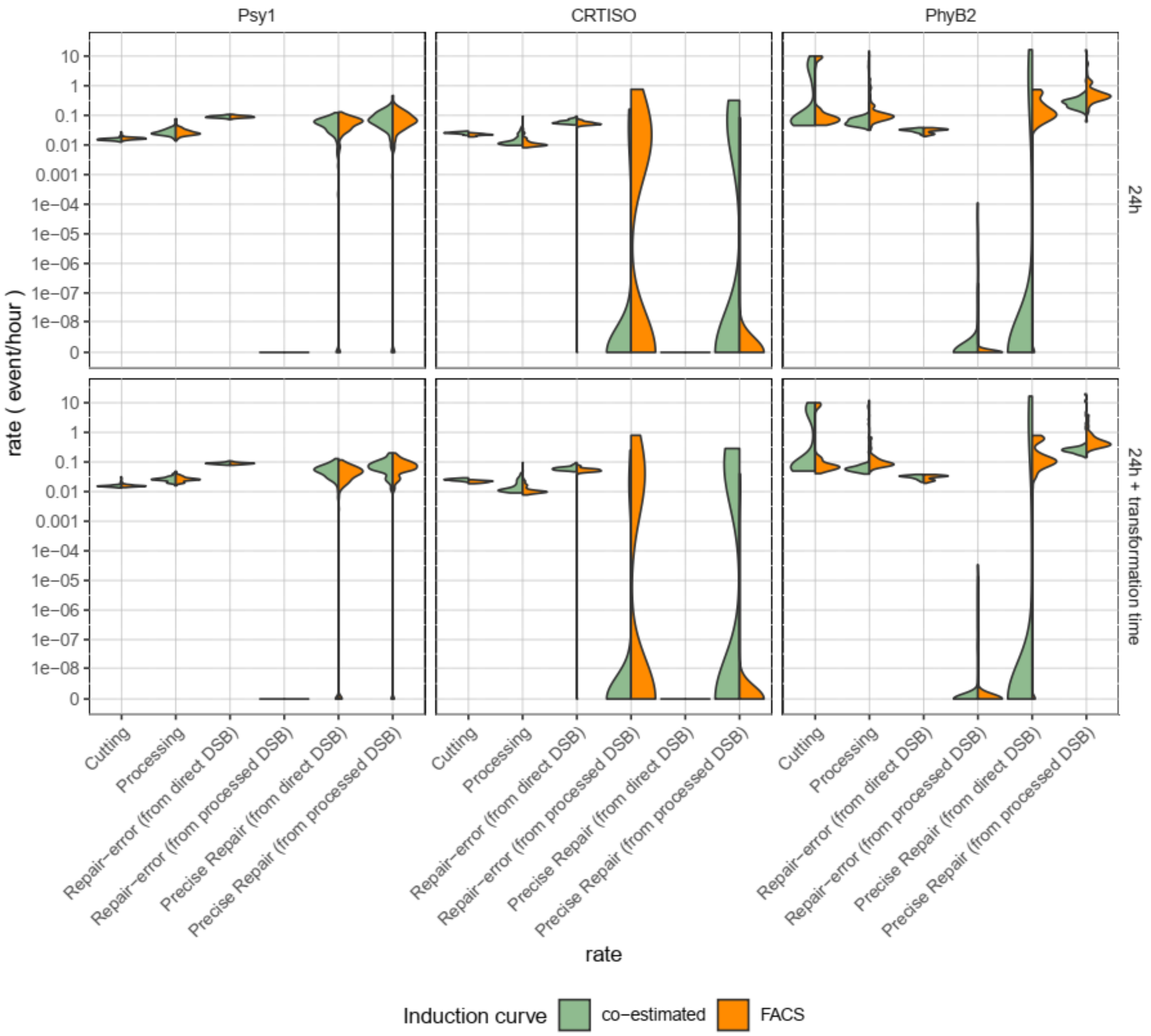

Figure S21. Comparison of estimates when co-estimating induction curve and repair parameters (green) or using the induction curve estimated from FACS data (orange) for the 4-state model in the 24-hour time course. Violin plots represent the bootstrap estimates for the two methods.

Target	Process	Rate Constant[a]	CI (bootstrap percentile)				Flow at 24h	CI (bootstrap percentile)				
			1%	5%	95%	99%		1%	5%	95%	99%	P.value
Psy1	K_{cut}	0.0165	0.014	0.0146	0.0186	0.0201	0.3257	0.3315	0.3315	0.3315	0.3315	<0.01
	$K_{processing}$	0.0254	0.0164	0.0206	0.0363	0.0431	/	0.0328	0.0328	0.0328	0.0328	<0.01
	E_{direct}	0.089	0.0777	0.0814	0.0992	0.1045	/	0.1279	0.1279	0.1279	0.1279	<0.01
	$E_{processed}$	0	0	0	0	0	/	0	0	0	0	1
	P_{direct}	0.0554	0	0.0203	0.0914	0.1032	/	0.1051	0.1051	0.1051	0.1051	0.01
	$P_{processed}$	0.0641	0	0.0221	0.1424	0.1968	/	0.0106	0.0106	0.0106	0.0106	0.01
	U	0.0253	0.0253	0.0253	0.0253	0.0253	/	/	/	/	/	<0.01
	r	85.9775	85.9775	85.9775	85.9775	85.9775	/	/	/	/	/	<0.01
	d	0.0026	0.0026	0.0026	0.0026	0.0026	/	/	/	/	/	<0.01
	Precise Repair	0.1195	0.0619	0.0773	0.1826	0.2358	/	0.1157	0.1157	0.1157	0.1157	<0.01
	Repair-error	0.089	0.0777	0.0814	0.0992	0.1045	/	0.1279	0.1279	0.1279	0.1279	<0.01
	Repair accuracy	0.5731	0.4153	0.4651	0.6722	0.7045	/	0.4749	0.4749	0.4749	0.4749	<0.01
CRTISO	K_{cut}	0.0223	0.019	0.0193	0.0249	0.0253	0.3916	0.4119	0.4119	0.4119	0.4119	<0.01
	$K_{processing}$	0.0101	0.0086	0.0092	0.0176	0.0321	/	0.0376	0.0376	0.0376	0.0376	<0.01
	E_{direct}	0.0537	0.0423	0.0466	0.0656	0.0708	/	0.1683	0.1683	0.1683	0.1683	<0.01
	$E_{processed}$	0	0	0	0.0709	0.1547	/	7.00E-04	7.00E-04	7.00E-04	7.00E-04	0.26
	P_{direct}	0	0	0	0	0	/	0	0	0	0	1
	$P_{processed}$	0	0	0	0	0.0066	/	0	0	0	0	0.98
	U	0.0253	0.0253	0.0253	0.0253	0.0253	/	/	/	/	/	<0.01
	r	85.9775	85.9775	85.9775	85.9775	85.9775	/	/	/	/	/	<0.01
	d	0.0026	0.0026	0.0026	0.0026	0.0026	/	/	/	/	/	<0.01
	Precise Repair	0	0	0	0	0.0066	/	0	0	0	0	0.99
	Repair-error	0.0537	0.0462	0.0483	0.1314	0.2109	/	0.169	0.169	0.169	0.169	<0.01
	Repair accuracy	0	0	0	0	0.058	/	0	0	0	0	0.99
CRTISO (-4bp Processed DSB)	K_{cut}	0.0736	0.0464	0.0508	9.8532	9.9998	25.4262	1.0469	1.0469	1.0469	1.0469	<0.01
	$K_{processing}$	0.0962	0.0418	0.0611	0.2199	0.861	/	0.3399	0.3399	0.3399	0.3399	<0.01
	E_{direct}	0.0322	0.0209	0.0223	0.0354	0.0375	/	0.1495	0.1495	0.1495	0.1495	<0.01
	$E_{processed}$	0	0	0	0	0	/	0	0	0	0	0.99
	P_{direct}	0.151	0	0.0558	47.4392	62.4427	/	0.3673	0.3673	0.3673	0.3673	0.01
	$P_{processed}$	0.4675	0.128	0.2443	1.3877	5.63	/	0.2988	0.2988	0.2988	0.2988	<0.01
	U	0.0253	0.0253	0.0253	0.0253	0.0253	/	/	/	/	/	<0.01
	r	85.9775	85.9775	85.9775	85.9775	85.9775	/	/	/	/	/	<0.01
	d	0.0026	0.0026	0.0026	0.0026	0.0026	/	/	/	/	/	<0.01
	Precise Repair	0.6185	0.3564	0.4043	48.8128	69.5168	/	0.6661	0.6661	0.6661	0.6661	<0.01
	Repair-error	0.0322	0.0209	0.0223	0.0354	0.0375	/	0.1495	0.1495	0.1495	0.1495	<0.01
	Repair accuracy	0.9505	0.9073	0.9208	0.9996	0.9997	/	0.8166	0.8166	0.8166	0.8166	<0.01
PhyB2	K_{cut}	0.0165	0.014	0.0146	0.0186	0.0201	0.3257	0.3315	0.3315	0.3315	0.3315	<0.01
	$K_{processing}$	0.0254	0.0164	0.0206	0.0363	0.0431	/	0.0328	0.0328	0.0328	0.0328	<0.01
	E_{direct}	0.089	0.0777	0.0814	0.0992	0.1045	/	0.1279	0.1279	0.1279	0.1279	<0.01
	$E_{processed}$	0	0	0	0	0	/	0	0	0	0	1
	P_{direct}	0.0554	0	0.0203	0.0914	0.1032	/	0.1051	0.1051	0.1051	0.1051	0.01
	$P_{processed}$	0.0641	0	0.0221	0.1424	0.1968	/	0.0106	0.0106	0.0106	0.0106	0.01
	U	0.0253	0.0253	0.0253	0.0253	0.0253	/	/	/	/	/	<0.01
	r	85.9775	85.9775	85.9775	85.9775	85.9775	/	/	/	/	/	<0.01
	d	0.0026	0.0026	0.0026	0.0026	0.0026	/	/	/	/	/	<0.01
	Precise Repair	0.1195	0.0619	0.0773	0.1826	0.2358	/	0.1157	0.1157	0.1157	0.1157	<0.01
	Repair-error	0.089	0.0777	0.0814	0.0992	0.1045	/	0.1279	0.1279	0.1279	0.1279	<0.01
	Repair accuracy	0.5731	0.4153	0.4651	0.6722	0.7045	/	0.4749	0.4749	0.4749	0.4749	<0.01

Table S11. Estimates of the Rates and Induction parameters for the 4-state model of DSB Repair for the 24-hour time course, with induction parameters fixed to those estimated from FACS data (U=0.0253, r=85.9775, d=0.0026, Fig. S9).

[a]Rates are reported as the number of events per molecule per hour. [b]The flow is reported as the proportion of molecules that experienced the specific event at the end of the experiment. [c]Confidence intervals (CI) are reported as the 1% and 99% percentiles of the estimates obtained from 100 stratified bootstraps of the data, while p.values as the proportion of bootstraps with value smaller or equal than 0 (one-sided test). When none of the 100 bootstraps had value equal to 0 we reported p.values as <0.01.

Table S12: Resources and Reagents

REAGENT or RESOURCE	SOURCE	IDENTIFIER
**Experimental models: Organisms/strains**		
M82		LA3475
**Chemicals, peptides, and recombinant proteins**		
pET-28b-Cas9-His	gift from Alex Schier [67]	Addgene plasmid # 47327<br>RRID:Addgene_47327
SpCas9 Protein	WSP–1 - Structural Proteomics Life Sciences Core Facilities	pET-28b-Cas9-His
$CaCl_2$	Sigma Aldrich	C1016-500G
NaCl	Bio-Lab Ltd	19030501
Mannitol	Sigma Aldrich	M9546-1KG
MES	Sigma Aldrich	M8250-100G
KCl	Sigma Aldrich	P5405-500G
$MgCl_2$	Sigma Aldrich	M9272
DEPC treated water	Biological Industries	01-852-1
Cellulase R10	Duchefa	YC-C8001-0010
Macerozyme	Duchefa	YM-M8002-0010
Probumin® Bovine Serum Albumin Life Science Grade	MILLIPORE	82-100-6
PEG 4000	Sigma Aldrich	25322-68-3
Buffer 3.1	New England Biolabs	Buffer 3.1
dNTPs	Thermo Fisher Scientific	R0181
Nuclease free water	Sigma Aldrich	W4502-1L
T4 Polynucleotide Kinase	New England BioLabs	M0201
T4 DNA polymerase	New England BioLabs	M0203
NEB Buffer 2	New England BioLabs	B7002S
dATP	New England BioLabs	N0440S
Klenow exo-	New England BioLabs	M0212
T4 ligase	New England BioLabs	M0202
2x Quick ligase Buffer	New England BioLabs	B6058S
Kapa HotStart Mix	Roche	07958927001
BEADS REAGENTS Sera-Mag™ magnetic carboxylate modified particles (Hydrophobic)	GE Healthcare Life Sciences-Cytiva	GE44152105050250Click or tap here to enter text.
Alt-R® CRISPR-Cas9 tracrRNA	Integrated DNA Technologies	1072534
EcoRI-HF	New England Biolabs	R3101
AanI-FD	New England Biolabs	FD2064
MspI	New England Biolabs	R0106
**Critical commercial assays**		
NucleoSpin™ Plant **II**	Machery-Nagel	15715543<br>740770.250
Qubit dsDNA HS Assay Kit	Thermo Fisher Scientific	Q32854
Tape Station:<br>High Sensitivity D1000 ScreenTap e,	Agilent Technologies	5067-5584

NovaSeq 6000 SP Reagent Kit v1.5 (300 cycles)	Illumina	20028400
NextSeq 500/550 Mid Output Kit v2.5 (300 Cycles)	Illumina	20024905
**Oligonucleotides**		
See Supplementary File 1 for a list of oligonucleotides		
**Software and algorithms**		
Bcl2fastq	https://support.illumina.com/sequencing/sequencing_software/bcl2fastq-conversion-software.html	bcl2fastq (RRID:SCR_01505)
BWA-MEM	https://github.com/bwa-mem2/bwa-mem2	BWA-MEM2 (RRID:SCR_02219)
picard/2.8.3	http://broadinstitute.github.io/picard/	Picard (RRID:SCR_00652)
GATK	https://software.broadinstitute.org/gatk/	GATK (RRID:SCR_00187)
fgbio	https://github.com/fulcrumgenomics/fgbio	Fulcrum Genomics
ea-utils (for fastq-join)	http://code.google.com/p/ea-utils/	ea-utils (RRID:SCR_00555)
Samtools	Li et al., 2009, http://samtools.sourceforge.net/	SAMTOOLS (RRID:SCR_00210)
Python	Python Programming Language (RRID:SCR_008394)	
Kinetic Model	This paper	Github
UMI-DSBseq Consensus sequence calling Bash scripts	This paper	Github
UMI-DSBseq Characterizing consensus sequences	This paper	Jupyter notebook Github
