# Supplementary material for: Uncovering the dynamics of precise repair at CRISPR/Cas9-induced double-strand breaks
Source: Nat Commun. 2024 Jun 14;15:5096. doi: 10.1038/s41467-024-49410-x (PMC11178868; doi:10.1038/s41467-024-49410-x)
Supplement: Supplementary file 2 — Reporting Summary [file 41467_2024_49410_MOESM2_ESM.pdf]

nature portfolio

Corresponding author(s): Avraham A. Levy

Last updated by author(s): Apr 18, 2024

Reporting Summary

Nature Portfolio wishes to improve the reproducibility of the work that we publish. This form provides structure for consistency and transparency in reporting. For further information on Nature Portfolio policies, see our Editorial Policies and the Editorial Policy Checklist.

Statistics

For all statistical analyses, confirm that the following items are present in the figure legend, table legend, main text, or Methods section.

n/a	Confirmed	
☐	☒	The exact sample size (*n*) for each experimental group/condition, given as a discrete number and unit of measurement
☐	☒	A statement on whether measurements were taken from distinct samples or whether the same sample was measured repeatedly
☐	☒	The statistical test(s) used AND whether they are one- or two-sided<br>*Only common tests should be described solely by name; describe more complex techniques in the Methods section.*
☐	☒	A description of all covariates tested
☐	☒	A description of any assumptions or corrections, such as tests of normality and adjustment for multiple comparisons
☐	☒	A full description of the statistical parameters including central tendency (e.g. means) or other basic estimates (e.g. regression coefficient) AND variation (e.g. standard deviation) or associated estimates of uncertainty (e.g. confidence intervals)
☐	☒	For null hypothesis testing, the test statistic (e.g. *F*, *t*, *r*) with confidence intervals, effect sizes, degrees of freedom and *P* value noted<br>*Give P values as exact values whenever suitable.*
☒	☐	For Bayesian analysis, information on the choice of priors and Markov chain Monte Carlo settings
☐	☒	For hierarchical and complex designs, identification of the appropriate level for tests and full reporting of outcomes
☐	☒	Estimates of effect sizes (e.g. Cohen's *d*, Pearson's *r*), indicating how they were calculated

Our web collection on statistics for biologists contains articles on many of the points above.

Software and code

Policy information about availability of computer code

Data collection	For generation of consensus sequences from raw Illumina basecalls:<br>bcl2fastq: https://support.illumina.com/sequencing/sequencing_software/bcl2fastq-conversion-software.html, RRID:SCR_01505<br>bwa/0.7.13: https://github.com/bwamem2/bwa-mem2 alignment; RRID:SCR_02219<br>picard/2.8.3: FastqToSam, MergeBamAlignment, SamToFastq; RRID:SCR_00652<br>fgbio/1.1.0: AnnotateBamWithUmis, GroupReadsByUmi, CallMolecularConsensusReads, https://github.com/fulcrumgenomics/fgbio,<br>ea-utils/1.1.2: fastq-join<br>A bash script detailing the pipeline can be found at https://github.com/daniebt/UMI-DSBseq.
Data analysis	Characterization and quantification of DSBs and repair types was achieved using a custom code written in python. Jupyter notebook and example are deposited in https://github.com/daniebt/UMI-DSBseq. All code for the kinetic modeling and statistical analysis has been deposited in GitHub and can be found at www.github.com/fabrimafe/DSBtimecourse.

For manuscripts utilizing custom algorithms or software that are central to the research but not yet described in published literature, software must be made available to editors and reviewers. We strongly encourage code deposition in a community repository (e.g. GitHub). See the Nature Portfolio guidelines for submitting code & software for further information.

Data

Policy information about availability of data

All manuscripts must include a data availability statement. This statement should provide the following information, where applicable:

- Accession codes, unique identifiers, or web links for publicly available datasets
- A description of any restrictions on data availability
- For clinical datasets or third party data, please ensure that the statement adheres to our policy

All work was done in Solanum Lycopersicum cv. M82 LA3475. BASH scripts for calling consensus sequences and Jupyter notebooks for characterizing DSB and repair types for UMI-DSBseq data can be found at https://github.com/daniebt/UMI-DSBseq. Kinetic model can be found at www.github.com/fabrimafe/DSBtimecourse. All our data are in a supplementary table. We have provide the minimum data set necessary to interpret, verify and extend the research in the article. We uploaded sequencing data as consensus joined fastq sequences, into NCBI, Project PRJNA1113841 and Flow Cytometry data to Zenodo (10.5281/zenodo.11255717)
Raw data are available upon request as specified in the Data Availability: ”Further information and requests for resources and reagents, as well as raw UMIDSBseq data should be directed to and will be fulfilled by the lead contact, avi.levy@weizmann.ac.il.” We pose no restriction to the data access.

Research involving human participants, their data, or biological material

Policy information about studies with human participants or human data. See also policy information about sex, gender (identity/presentation), and sexual orientation and race, ethnicity and racism.

Reporting on sex and gender	N/A
Reporting on race, ethnicity, or other socially relevant groupings	N/A
Population characteristics	N/A
Recruitment	N?A
Ethics oversight	N/A

Note that full information on the approval of the study protocol must also be provided in the manuscript.

Field-specific reporting

Please select the one below that is the best fit for your research. If you are not sure, read the appropriate sections before making your selection.

☒ Life sciences ☐ Behavioural & social sciences ☐ Ecological, evolutionary & environmental sciences

For a reference copy of the document with all sections, see nature.com/documents/nr-reporting-summary-flat.pdf

Life sciences study design

All studies must disclose on these points even when the disclosure is negative.

Sample size	CRTISO target time-course: 7 time-points, n=2 independent replicates per time-point, 14 samples total<br>CRTISO target control time-course: 7 time-points, n=2 independent replicates per time-point, 14 samples total<br>Psy1 target time-course: 7 time-points, n=2 independent replicates per time-point, 14 samples total<br>Psy1 target control time-course: 7 time-points, n=2 independent replicates per time-point, 14 samples total<br>PhyB2 target time-course: 7 time-points, n=2 independent replicates per time-point, 14 samples total<br>PhyB2 target control time-course: 7 time-points, n=2 independent replicates per time-point, 14 samples total<br>CRTISO in vitro cleavage: n=3 independent replicates<br>Psy1 in vitro cleavage: n=3 independent replicates<br>PhyB2 in vitro cleavage: n=3 independent replicates
Data exclusions	No data from the reported experiments was excluded.
Replication	The findings were confirmed, independently, at 3 loci targeting similar genomic regions (exons). The observed measurements were reproducible between the independent replicates in the majority of sampling points, in all time-courses.
Randomization	randomization was not applicable during the experimental setup due to the structured nature i.e. single targets and single time-points
Blinding	Blinding was not possible since the analysis focused on specific targets and time-points

Reporting for specific materials, systems and methods

We require information from authors about some types of materials, experimental systems and methods used in many studies. Here, indicate whether each material, system or method listed is relevant to your study. If you are not sure if a list item applies to your research, read the appropriate section before selecting a response.

Materials & experimental systems

n/a	Involved in the study	
☒	☐	Antibodies
☒	☐	Eukaryotic cell lines
☒	☐	Palaeontology and archaeology
☒	☐	Animals and other organisms
☒	☐	Clinical data
☒	☐	Dual use research of concern
☐	☒	Plants

Methods

n/a	Involved in the study	
☒	☐	ChIP-seq
☐	☒	Flow cytometry
☒	☐	MRI-based neuroimaging

Dual use research of concern

Policy information about dual use research of concern

Hazards

Could the accidental, deliberate or reckless misuse of agents or technologies generated in the work, or the application of information presented in the manuscript, pose a threat to:

No	Yes	
☒	☐	Public health
☒	☐	National security
☒	☐	Crops and/or livestock
☒	☐	Ecosystems
☒	☐	Any other significant area

Experiments of concern

Does the work involve any of these experiments of concern:

No	Yes	
☒	☐	Demonstrate how to render a vaccine ineffective
☒	☐	Confer resistance to therapeutically useful antibiotics or antiviral agents
☒	☐	Enhance the virulence of a pathogen or render a nonpathogen virulent
☒	☐	Increase transmissibility of a pathogen
☒	☐	Alter the host range of a pathogen
☒	☐	Enable evasion of diagnostic/detection modalities
☒	☐	Enable the weaponization of a biological agent or toxin
☒	☐	Any other potentially harmful combination of experiments and agents

Plants

Seed stocks	Solanum Lycopersicum M82 LA3475
Novel plant genotypes	N/A
Authentication	N/A

Flow Cytometry

Plots

Confirm that:

- [x] The axis labels state the marker and fluorochrome used (e.g. CD4-FITC).
- [x] The axis scales are clearly visible. Include numbers along axes only for bottom left plot of group (a 'group' is an analysis of identical markers).
- [x] All plots are contour plots with outliers or pseudocolor plots.
- [x] A numerical value for number of cells or percentage (with statistics) is provided.

Methodology

Sample preparation	Nuclei were isolated from Tomato protoplasts
Instrument	FACSAria III cell sorter (GB biosciences)
Software	BD FACSDiva™ Software
Cell population abundance	The number of nuclei ranged between 9000-22000.
Gating strategy	Gating was designed to count only single and round nuclei based on the measured forward scatter (FSC), side scatter (SSC), and DAPI fluorescence.

- [x] Tick this box to confirm that a figure exemplifying the gating strategy is provided in the Supplementary Information.
