# Supplementary material for: Uncovering the dynamics of precise repair at CRISPR/Cas9-induced double-strand breaks
Source: Nat Commun. 2024 Jun 14;15:5096. doi: 10.1038/s41467-024-49410-x (PMC11178868; doi:10.1038/s41467-024-49410-x)
Supplement: Supplementary file 3 — Description of Additional Supplementary Files [file 41467_2024_49410_MOESM3_ESM.pdf]

Supplementary Data 1:
Oligonucleotides used in this study
