# Supplementary material for: Uncovering the dynamics of precise repair at CRISPR/Cas9-induced double-strand breaks
Source: Nat Commun. 2024 Jun 14;15:5096. doi: 10.1038/s41467-024-49410-x (PMC11178868; doi:10.1038/s41467-024-49410-x)
Supplement: Supplementary file 5 — Supplementary data 1 [file 41467_2024_49410_MOESM5_ESM.pdf]

Supplementary File 1: Oligonucleotides used in this study

**Oligonucleotides**		
UMI-DSBseq Adaptor sequences		
Adaptor Short Tail	GATCGGAAGAGCGGGGACTATTTGC	IDT Ultramer DNA Oligos
Adaptor long tail UMI barcode i7_1	CAAGCAGAAGACGGCATACGAGATNNNNNNNNNACGATCAGGTGACTGGAGTTCAGACGTGTGCTCTTCCGATC*T (*denotes phosphorothioate bond)	IDT Ultramer DNA Oligos
Adaptor long tail UMI barcode i7_2	CAAGCAGAAGACGGCATACGAGATNNNNNNNNNTCGAGAGTGTGACTGGAGTTCAGACGTGTGCTCTTCCGATC*T (*denotes phosphorothioate bond)	IDT Ultramer DNA Oligos
Adaptor long tail UMI barcode i7_3	CAAGCAGAAGACGGCATACGAGATNNNNNNNNNCTAGCTCAGTGACTGGAGTTCAGACGTGTGCTCTTCCGATC*T (*denotes phosphorothioate bond)	IDT Ultramer DNA Oligos
Adaptor long tail UMI barcode i7_4	CAAGCAGAAGACGGCATACGAGATNNNNNNNNNATCGTCTCGTGACTGGAGTTCAGACGTGTGCTCTTCCGATC*T (*denotes phosphorothioate bond)	IDT Ultramer DNA Oligos
Adaptor long tail UMI barcode i7_5	CAAGCAGAAGACGGCATACGAGATNNNNNNNNNTCGACAAGGTGACTGGAGTTCAGACGTGTGCTCTTCCGATC*T (*denotes phosphorothioate bond)	IDT Ultramer DNA Oligos
Adaptor long tail UMI barcode i7_6	CAAGCAGAAGACGGCATACGAGATNNNNNNNNNCCTTGGAAGTGACTGGAGTTCAGACGTGTGCTCTTCCGATC*T (*denotes phosphorothioate bond)	IDT Ultramer DNA Oligos
Adaptor long tail UMI barcode i7_7	CAAGCAGAAGACGGCATACGAGATNNNNNNNNNATCATGCGGTGACTGGAGTTCAGACGTGTGCTCTTCCGATC*T (*denotes phosphorothioate bond)	IDT Ultramer DNA Oligos
Adaptor long tail UMI barcode i7_8	CAAGCAGAAGACGGCATACGAGATNNNNNNNNNTGTTCCGTGTGACTGGAGTTCAGACGTGTGCTCTTCCGATC (*denotes phosphorothioate bond)	IDT Ultramer DNA Oligos
Adaptor long tail UMI barcode i7_9	CAAGCAGAAGACGGCATACGAGATNNNNNNNNNATTAGCCGGTGACTGGAGTTCAGACGTGTGCTCTTCCGATC*T (*denotes phosphorothioate bond)	IDT Ultramer DNA Oligos
Adaptor long tail UMI barcode i7_10	CAAGCAGAAGACGGCATACGAGATNNNNNNNNNCGATCGATGTGACTGGAGTTCAGACGTGTGCTCTTCCGATC*T (*denotes phosphorothioate bond)	IDT Ultramer DNA Oligos
Adaptor long tail UMI barcode i7_11	CAAGCAGAAGACGGCATACGAGATNNNNNNNNNGATCTTGCGTGACTGGAGTTCAGACGTGTGCTCTTCCGATC*T (*denotes phosphorothioate bond)	IDT Ultramer DNA Oligos
Adaptor long tail UMI barcode i7_12	CAAGCAGAAGACGGCATACGAGATNNNNNNNNNAGGATAGCGTGACTGGAGTTCAGACGTGTGCTCTTCCGATC*T (*denotes phosphorothioate bond)	IDT Ultramer DNA Oligos
Adaptor long tail UMI barcode i7_13	CAAGCAGAAGACGGCATACGAGATNNNNNNNNNGTAGCGTAGTGACTGGAGTTCAGACGTGTGCTCTTCCGATC*T (*denotes phosphorothioate bond)	IDT Ultramer DNA Oligos
Adaptor long tail UMI barcode i7_14	CAAGCAGAAGACGGCATACGAGATNNNNNNNNNAGAGTCCAGTGACTGGAGTTCAGACGTGTGCTCTTCCGATC*T (*denotes phosphorothioate bond)	IDT Ultramer DNA Oligos
**Primer Sequences**		
P5 Target specific primer for PhyB2<br>P5 PhyB2 exon1 DSB2 Rev	AATGATACGGCGACCACCGAGATCTACACTCTTTCCCTACACGACGCTCTTCCGATCTNNNGTGGGTGAGTCTCGGAGAAG	This paper
P5 Target specific primer for Psy1<br>P5 Psy1-1 HTseq F	AATGATACGGCGACCACCGAGATCTACACTCTTTCCCTACACGACGCTCTTCCGATCTNNNGGTTTGCCTGTCTGTGGTCT	Filler-Hayut et al., 2017
P5 Target specific primer for CRTISO<br>*P5 CRT_e4_DSB F*	AATGATACGGCGACCACCGAGATCTACACTCTTTCCCTACACGACGCTCTTCCGATCTNNNGATGGTCCTGAGTGTTTGCC	This paper
P7 amplification and enrichment primer	CAAGCAGAAGACGGCATACGAGAT	Blecher-Gonenet al.,2013
P5 enrichment primer with barcode	AATGATACGGCGACCACCGAGATCTACAC**NNNNNNNN**ACACTCTTTCCCTACACGAC	Adapted from Blecher-Gonenet al.,2013

**PCR_DIRECT**		
Psy1_Direct_PCR_F	TTCCCTACACGACGCTCTTCCGATCTGGTTTGCCTGTCTGTGGTCT	
Psy1_Direct_PCR_R	GTTCAGACGTGTGCTCTTCCGATCTCCATGAAACTTGTCCCATTTG	
I5_index_Direct_PCR_F	AATGATACGGCGACCACCGAGATCTACACNNNNNNNNACACTCTTTCCCTACACGAC	
I7_index_Direct_PCR_R	CAAGCAGAAGACGGCATACGAGATNNNNNNNNGTGACTGGAGTTCAGACGTGTGCTCTTC	
**crRNA**		
CRTISO Alt-R® CRISPR-Cas9 crRNA	GCGATGCTACCAGCATTCTG	Dahan-Meir et al., 2018
Psy1 Alt-R® CRISPR-Cas9 crRNA	GAATGTCTGTTGCCTTGTTA	Filler-Hayut et al., 2017
PhyB2 Alt-R® CRISPR-Cas9 crRNA	GGCCTGCATAAGGAATTCAC	This paper

Target Sequences

Target	***Amplicon***	***Restriction enzyme***
CRTISO	TATTTAAGTGAAGAATGGTAAAGGGGAGGGGCCATTATCCCCGAGTTTTGAGCACTATTGATGGTCCTGAGTGTTTGCCCTCGGTCATCAAAAAAATTTAAGGAGTCATCTTTCACGCTGATGTGTGCAGCGCGCGACGTGCTTAATTATCCTACCGTAGAATCTTAATTTATGCCATCATTATTCATTACAGCCTACTATTTGCCCCAGAATGCTGGTAGCATCGCTCGGAAGTATATAAGAGATCCTGGGTTGCTGTCTTTTATAGATGCAGAG	EcoRI-HF
Psy1	GGTTTGCCTGTCTGTGGTCTTTTTATAATCTTTTTCTACAGAAGAGAAAGTGGGTAATTTTGTTTGAGAGTGGAAATATTCTCTAGTGGGAATCTACTAGGAGTAATTTATTTTCTATAAACTAAGTAAAGTTTGGAAGGTGACAAAAAGAAAGACAAAAATCTTGGAATTGTTTTAGACAACCAAGGTTTTCTTGCTCAGAATGTC**TGTT**GCCT**TGTT**ATGGGTTGTTTCTCCTTGTGACGTCTCAAATGGGACAAGTTTCATGGAATCAGTCCG	MspI
PhyB2	GTGGGTGAGTCTCGGAGAAGCATGTCACATAACACTGTCTGTGTCCTCAAAACCCGCTTTTCAGCCAACTGCGATGCCAATTGTAATTCCATGTTTAGTTGGAGCCCAAAGGCCTGCATAAGGAATTCACAGGCATAACGAAGGGGGAAAGGAATGAACCGAGCTGAACTGTGGTGCCCAACAACCAAACCCCATAATCTCATTGCATTTCGGCCTCCAACAACTTCATCATCATTTCCATTTA	AanI-FD

*Microhomologies at *Psy1* are underlined and bolded The yellow highlighted bases represents the crRNA sequence. The green highlighted bases is the PAM.
